# Multiple Common Susceptibility Variants near BMP Pathway Loci *GREM1*, *BMP4*, and *BMP2* Explain Part of the Missing Heritability of Colorectal Cancer

**DOI:** 10.1371/journal.pgen.1002105

**Published:** 2011-06-02

**Authors:** Ian P. M. Tomlinson, Luis G. Carvajal-Carmona, Sara E. Dobbins, Albert Tenesa, Angela M. Jones, Kimberley Howarth, Claire Palles, Peter Broderick, Emma E. M. Jaeger, Susan Farrington, Annabelle Lewis, James G. D. Prendergast, Alan M. Pittman, Evropi Theodoratou, Bianca Olver, Marion Walker, Steven Penegar, Ella Barclay, Nicola Whiffin, Lynn Martin, Stephane Ballereau, Amy Lloyd, Maggie Gorman, Steven Lubbe, The COGENT Consortium, The CORGI Collaborators, The EPICOLON Consortium, Bryan Howie, Jonathan Marchini, Clara Ruiz-Ponte, Ceres Fernandez-Rozadilla, Antoni Castells, Angel Carracedo, Sergi Castellvi-Bel, David Duggan, David Conti, Jean-Baptiste Cazier, Harry Campbell, Oliver Sieber, Lara Lipton, Peter Gibbs, Nicholas G. Martin, Grant W. Montgomery, Joanne Young, Paul N. Baird, Steven Gallinger, Polly Newcomb, John Hopper, Mark A. Jenkins, Lauri A. Aaltonen, David J. Kerr, Jeremy Cheadle, Paul Pharoah, Graham Casey, Richard S. Houlston, Malcolm G. Dunlop

**Affiliations:** 1 Wellcome Trust Centre for Human Genetics, University of Oxford, Oxford, United Kingdom; 2 Section of Cancer Genetics, Institute of Cancer Research, Sutton, United Kingdom; 3 Colon Cancer Genetics Group, Institute of Genetics and Molecular Medicine, University of Edinburgh and Medical Research Council Human Genetics Unit, Edinburgh, United Kingdom; 4 The University of Edinburgh Medical School, Edinburgh, United Kingdom; 5 Department of Statistics, University of Oxford, Oxford, United Kingdom; 6 Galician Public Foundation of Genomic Medicine (FPGMX), Centro de Investigacion Biomedica en Red de Enfermedades Raras (CIBERER), Genomics Medicine Group, Hospital Clinico, Santiago de Compostela, University of Santiago de Compostela, Galicia, Spain; 7 Department of Gastroenterology, Hospital Clinic, CIBERehd, IDIBAPS, University of Barcelona, Barcelona, Catalonia, Spain; 8 Translational Genomics Research Institute, Phoenix, Arizona, United States of America; 9 Department of Preventive Medicine, University of Southern California, Los Angeles, California, United States of America; 10 Public Health Sciences, University of Edinburgh, Edinburgh, United Kingdom; 11 Ludwig Colon Cancer Initiative Laboratory, Ludwig Institute for Cancer Research, Royal Melbourne Hospital, Parkville, Australia; 12 Genetic and Molecular Epidemiology Laboratories, Queensland Institute of Medical Research, Brisbane, Australia; 13 Familial Cancer Laboratory, Queensland Institute of Medical Research, Brisbane, Australia; 14 Centre for Eye Research Australia, University of Melbourne, Melbourne, Australia; 15 Samuel Lunenfeld Research Institute, Mount Sinai Hospital, Toronto, Canada; 16 Fred Hutchinson Cancer Research Center, Seattle, Washington, United States of America; 17 Centre for Molecular, Environmental, Genetic, and Analytic Epidemiology, The University of Melbourne, Australia; 18 Department of Medical Genetics, Genome-Scale Biology Research Program, Biomedicum Helsinki, University of Helsinki, Helsinki, Finland; 19 Department of Clinical Pharmacology, University of Oxford, Oxford, United Kingdom; 20 Institute of Medical Genetics, School of Medicine, Cardiff University, Cardiff, United Kingdom; 21 Cancer Research UK Laboratories, Strangeways Research Laboratory, Department of Oncology, University of Cambridge, Cambridge, United Kingdom; Georgia Institute of Technology, United States of America

## Abstract

Genome-wide association studies (GWAS) have identified 14 tagging single nucleotide polymorphisms (tagSNPs) that are associated with the risk of colorectal cancer (CRC), and several of these tagSNPs are near bone morphogenetic protein (BMP) pathway loci. The penalty of multiple testing implicit in GWAS increases the attraction of complementary approaches for disease gene discovery, including candidate gene- or pathway-based analyses. The strongest candidate loci for additional predisposition SNPs are arguably those already known both to have functional relevance and to be involved in disease risk. To investigate this proposition, we searched for novel CRC susceptibility variants close to the BMP pathway genes *GREM1* (15q13.3), *BMP4* (14q22.2), and *BMP2* (20p12.3) using sample sets totalling 24,910 CRC cases and 26,275 controls. We identified new, independent CRC predisposition SNPs close to *BMP4* (rs1957636, *P* = 3.93×10^−10^) and *BMP2* (rs4813802, *P* = 4.65×10^−11^). Near *GREM1*, we found using fine-mapping that the previously-identified association between tagSNP rs4779584 and CRC actually resulted from two independent signals represented by rs16969681 (*P* = 5.33×10^−8^) and rs11632715 (*P* = 2.30×10^−10^). As low-penetrance predisposition variants become harder to identify—owing to small effect sizes and/or low risk allele frequencies—approaches based on informed candidate gene selection may become increasingly attractive. Our data emphasise that genetic fine-mapping studies can deconvolute associations that have arisen owing to independent correlation of a tagSNP with more than one functional SNP, thus explaining some of the apparently missing heritability of common diseases.

## Author Summary

Genome-wide association studies (GWAS) have identified several colorectal cancer (CRC) susceptibility polymorphisms near genes that encode proteins in the bone morphogenetic protein (BMP) pathway. However, most of the inherited susceptibility to CRC remains unexplained. We investigated three of the best candidate BMP genes (*GREM1*, *BMP4*, and *BMP2*) for additional polymorphisms associated with CRC. By extensive validation of polymorphisms with only modest evidence of association in the initial phases of the GWAS, we identified new, independent CRC predisposition polymorphisms close to *BMP4* (rs1957636) and *BMP2* (rs4813802). Near *GREM1*, we used additional genotyping around the GWAS-identified polymorphism rs4779584 to demonstrate two independent signals represented by rs16969681 and rs11632715. Common genes with modest effects on disease risk are becoming harder to identify, and approaches based on informed candidate gene selection may become increasingly attractive. In addition, genetic fine mapping around polymorphisms identified in GWAS can deconvolute associations which have arisen owing to two independent functional variants. These types of study can identify some of the apparently missing heritability of common disease.

## Introduction

Genome-wide association (GWA) studies of colorectal cancer (CRC) have so far identified 14 common, low-risk susceptibility variants [1]. Of these 14 variants, 3 are close to loci that are secreted members of the bone morphogenetic protein (BMP) signalling pathway: *GREM1* (rs4779584); *BMP4* (rs4444235); and *BMP2* (rs961253). In the colon, GREM1 is one of several BMP antagonists produced by sub-epithelial myofibroblasts (ISEMFs). GREM1 binds to and inactivates the ligands BMP2 and BMP4 that are primarily produced by inter-cryptal stromal cells.

Our GWA studies have utilised a primary phase of genome-wide typing of tagging single nucleotide polymorphisms (tagSNPs), followed by larger validation phases of those SNPs with the strongest signals of association. We have previously used relatively stringent statistical thresholds to take SNPs forward into the final validation phases [1]. Whilst such a design has been cost-effective, the use of a lower threshold may have led to the discovery of more CRC SNPs, albeit at the cost of a relatively high type I error rate. One means of reducing false positives might be to select SNPs using a less stringent threshold where there is *a priori* evidence for candidacy. We reasoned that the best candidate loci were those already identified as harbouring CRC risk alleles. Of those 14 loci, we prioritised *GREM1*, *BMP2* and *BMP4* for further analysis owing to their strongly-related functions.

The GWA studies had identified a single tagSNP associated with CRC risk close to each of *GREM1*, *BMP2* and *BMP4* [1]. Examination of the regions around these genes in public databases such as HapMap (http://www.hapmap.org/) showed in all cases that the coding sequence and predicted surrounding regulatory regions were present within more than one linkage disequilibrium (LD) block. For each of the 3 genes, therefore, it was possible that there were additional genetic determinants of CRC risk, independent of the already-identified SNPs. We proceeded to test this hypothesis in large sets of CRC cases and controls of European origin.

## Results

### The rs4779584 CRC signal results from two independent SNPs close to *GREM1*

**Figure 1:**
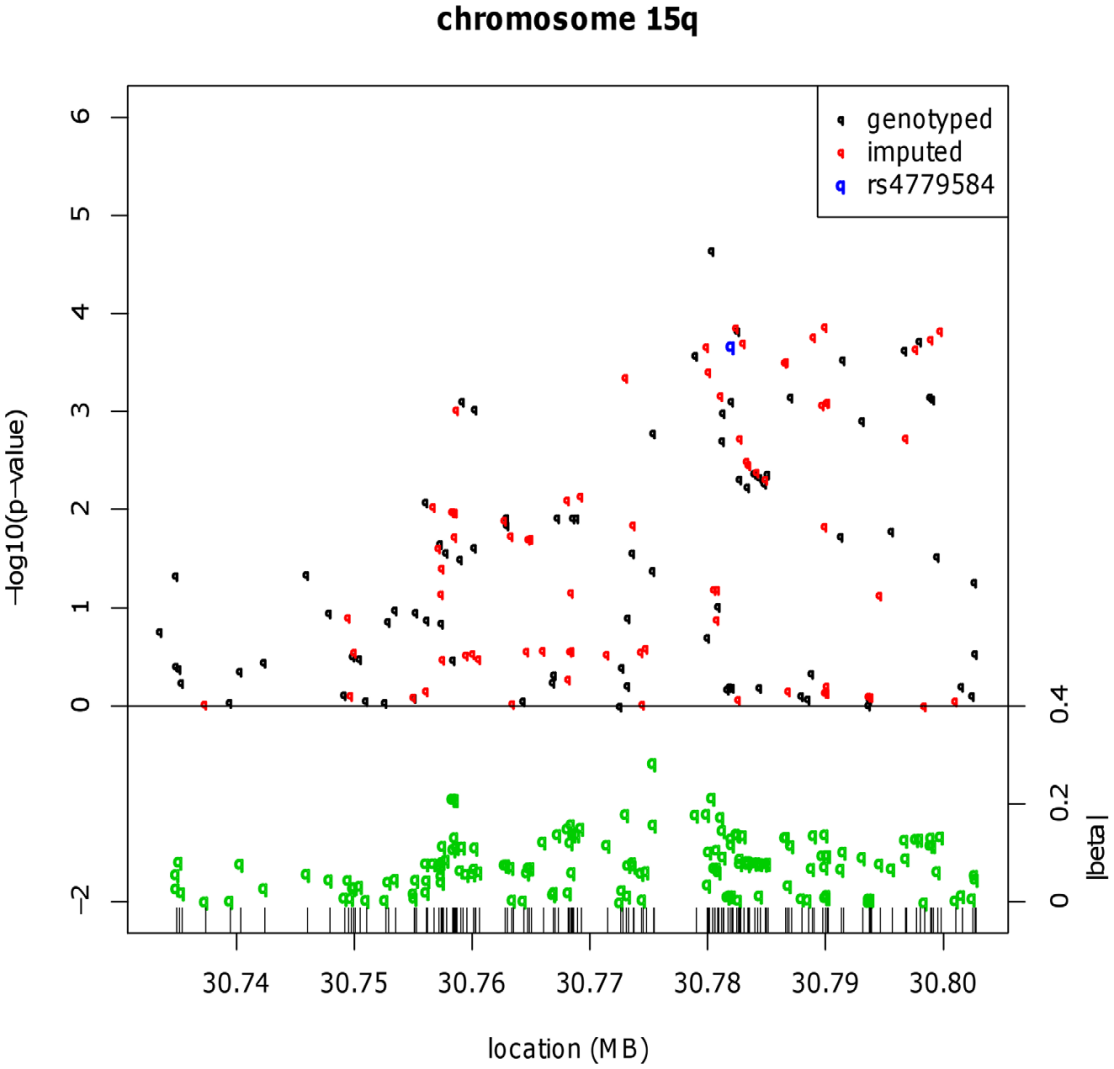
Fine mapping around the known CRC risk SNP close to *GREM1* (15q13.3). Results for meta-analysis of UK2 and Scotland2 are shown. Both significance of association (−log_10_(P)) and effect size (β) are presented. The original CRC-associated tagSNPs are shown in blue. The SNP with the clearly strongest association signal is the genotyped SNP rs16969681.

In order to refine the location of CRC-associated functional variation close to the *GREM1*, *BMP4* and *BMP2* loci, we genotyped 442 SNPs close to rs4779584, rs4444235 and rs961253 in 4,878 CRC cases and 4,914 controls from the UK2 and Scotland2 sample sets and imputed other SNPs within these regions. No significant localisation of a functional variant was achieved for rs4444235 or rs961253 (Figure S1), but at *GREM1*, rs16969681 (chr15:30,780,403 bases) had a notably stronger signal of association than rs4779584 (pairwise LD: r^2^ = 0.18, D′ = 0.70) (Figure 1 and Figure S2). We genotyped rs16969681 in additional independent CRC case-control series (UK1, UK4, VQ58, Helsinki, Cambridge and EPICOLON; see Methods). After combined analysis, a significant association between the minor allele at rs16969681 and CRC risk was seen (*P* = 5.33×10^−8^; Table 1). Unconditional logistic regression analysis, incorporating sample series as a co-variate, showed that rs16969681 was more strongly associated with CRC than rs4779584, but that the signals were non-independent (for rs16969681, OR = 1.16, 95% CI 1.07–1.25, *P* = 1.91×10^−4^; for rs4779584, OR = 1.08, 95% CI 1.02–1.14, *P* = 5.27×10^−3^). Akaike information criteria metrics for rs16969681 and rs4779584 respectively were 25608 and 25922, consistent with a superior fit of the risk model incorporating the former SNP. Intriguingly, we found that rs16969681 maps to a site of open chromatin in *GREM1*-expressing CRC cell lines, raising the possibility that it may be directly functional (Figure S3).

**Table 1:** Genotype counts and statistics of association at rs16969681, rs11632715, and rs4779584.

	Sample series		Case genotypes			Control genotypes					Odds ratio
			TT	TC	CC	TT	TC	CC	freqT(cases)	freqT(controls)	
rs16969681	1	UK2	29	500	2335	28	399	2428	0.097	0.080	1.247
z = 5.44	2	Scotland2	16	337	1653	10	293	1754	0.092	0.076	1.230
P_overall_ = 5.33×10^−8^	3	UK1	12	183	746	4	145	773	0.110	0.083	1.366
OR = 1.181	4	VQNBS	8	235	1074	26	491	2418	0.095	0.093	1.033
95%CI 1.113–1.254	5	EPICOLON	13	313	1234	14	229	1025	0.109	0.101	1.081
P_het_ = 0.013, I2 = 60.8%	6	Helsinki	14	189	742	8	104	726	0.115	0.072	1.682
	7	UK4	6	95	482	6	180	862	0.092	0.092	1.002
P_replicationphase_ = 2.73×10^−4^	8	Cambridge	21	370	1852	41	279	1818	0.092	0.084	1.097

			TT	TC	CC	TT	TC	CC	freqT(cases)	freqT(controls)	
rs4779584	1	UK2	155	934	1762	102	857	1858	0.218	0.188	1.203
z = 6.30	2	Scotland2	84	603	1276	72	608	1332	0.196	0.187	1.063
P_overall_ = 2.98×10^−10^	3	UK1	52	316	533	30	288	611	0.233	0.187	1.319
OR = 1.145	4	VQNBS	81	564	1155	102	797	1601	0.202	0.200	1.009
95%CI 1.098–1.195	5	EPICOLON	61	434	878	51	396	934	0.202	0.180	1.154
P_het_ = 0.056, I2 = 45.8%	6	Helsinki	88	362	378	69	352	418	0.325	0.292	1.167
	7	UK4	33	174	361	27	241	426	0.211	0.213	0.992
	8	Scotland1	55	331	591	39	286	676	0.226	0.182	1.312
	9	CCFR	58	412	716	32	319	647	0.223	0.192	1.206
	10	Australia	22	149	269	17	136	285	0.219	0.194	1.167

			AA	AG	GG	AA	AG	GG	freqA(cases)	freqA(controls)	
rs11632715	1	UK2	719	1415	718	600	1450	768	0.500	0.470	1.128
z = 6.34	2	Scotland2	475	999	532	438	1040	579	0.486	0.466	1.084
P_overall_ = 2.30×10^−10^	3	UK1	244	469	207	222	457	250	0.520	0.485	1.151
OR = 1.116	4	VQNBS	446	913	440	567	1252	679	0.502	0.478	1.101
95%CI 1.079–1.155	5	EPICOLON	308	684	401	238	651	378	0.467	0.445	1.092
P_het_ = 0.997, I2 = 0.0%	6	Helsinki	182	488	272	150	409	276	0.452	0.425	1.119
	7	UK4	150	281	141	222	534	285	0.508	0.470	fs1.165
P_replicationphase_ = 1.79×10^−7^	8	Scotland1	238	486	256	219	496	287	0.491	0.466	1.104
	9	CCFR	286	592	311	202	510	287	0.489	0.457	1.137
	10	Australia	106	226	105	88	231	119	0.501	0.465	1.158

All data sets in which rs16969681 and/or rs11632715 were genotyped are shown. The sample sets genotyped for the SNPs near *GREM1* are overlapping, but non-identical, largely because rs11632715 and rs4779584 (but not rs16969681) are present on the proprietary Illumina genome-wide arrays, and also because the Cambridge data set was additionally genotyped for rs16969681. In addition to the overall association test statistics, the P value for the replication phase (excluding UK2 and Scotland2) is shown for rs16969681 and rs11632715. Although there is considerable overlap, the sample sets genotyped here differ somewhat from those typed for the *BMP2* and *BMP4* SNPs. These differences result entirely from sample and data availability and practical issues of genotyping, including the following: GWAS data but not samples were available from some data sets, so that SNPs such as rs16969681 could not be genotyped in those sample sets; the 1958 Birth Cohort samples were not available at the time of genotyping rs16969681; and for some sample sets, DNA quantity was limiting.

Haplotype risk analysis (Table S2) provided evidence that rs16969681 alleles do not capture all the CRC risk associated with rs4779584. In brief, data from UK2 and Scotland2 showed that the risk alleles at rs16969681 and rs4779584 were defined by a TGGTC haplotype at rs16969681-rs16969862-rs12594722-rs4779584-rs9888701. The TT rs16969681-rs4779584 haplotype was at a frequency of 0.063 in cases and 0.052 in controls (*P* = 6.29×10^−5^). However, there appeared to be a residual effect of the T allele at rs4779584, since there was also an elevated risk associated with the CT rs16969681-rs4779584 haplotype (*P* = 0.026).

We therefore tested the hypothesis that rs4779584 tags two independent risk SNPs at *GREM1*. We used reverse stepwise logistic regression to search the set of *GREM1* SNPs genotyped in the UK2 and Scotland2 samples (Table S1) for associations that were independent of rs16969681 genotype and that captured the residual rs4779584 signal. This analysis led to elimination of rs4779584 from the regression model and identification of a model in which only rs16969681 (*P* = 1.04×10^−4^) and another SNP, rs11632715 (*P* = 1.00×10^−3^), produced independent signals. rs11632715 (chr15:30,791,539) is in low LD with rs16969681 (r^2^ = 0.009, D′ = 0.31) and modest LD with rs4779584 (r^2^ = 0.18, D′ = 0.90; Figure S2). Through genotyping of additional case-control series, we showed that rs11632715 was significantly associated with CRC risk (*P* = 2.30×10^−10^; Table 1). Unconditional logistic regression in the 21,139 samples typed for both rs11632715 and rs16969681 provided confirmatory evidence of the independence of the signals (for rs16969681, *P* = 1.84×10^−6^ and for rs11632715, *P* = 6.36×10^−7^); these associations were of very similar magnitude to those obtained when each SNP was analysed individually in those sample sets (Figure S2). Incorporation of rs4779584 into the logistic regression model showed that this SNP had a weaker effect than that of either rs16969681 or rs11632715 and did not significantly improve the model fit (Table S3). Inspection of the region containing rs4779584, rs16969681 and rs11632715 (Figure S2) showed that rs4779584 lay within a recombination hotspot. This finding was consistent with our discovery that rs4779584 tags two independent functional variants that are, in turn, tagged by rs16969681 and rs11632715.

### Identification of additional, independent CRC susceptibility SNPs near *BMP4* and *BMP2*

**Figure 2:**
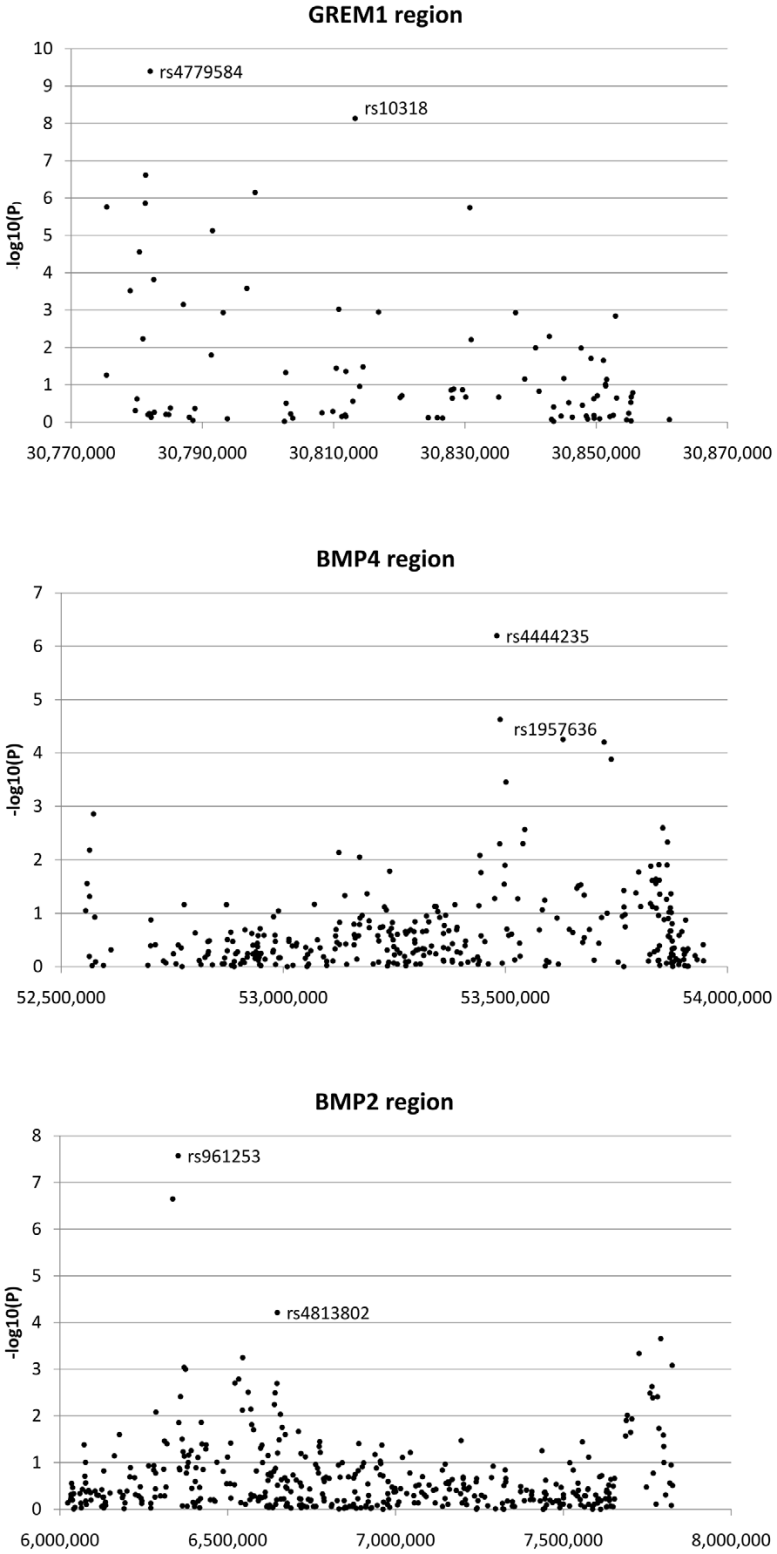
Search for additional colorectal cancer susceptibility SNPs near *GREM1*, *BMP4*, and *BMP2*. Association signals from discovery phase around *GREM1*, *BMP4* and *BMP2* are shown. For *GREM1*, the labelled SNPs are highyl correlated tagSNPs originally reported as associated with CRC; these signals are non-independent. For *BMP4* and *BMP2*, the labelled SNPs are the original tagSNPs and the subsequently proven new signals at rs1957636 and rs4813802 respectively.

The regions analysed for fine mapping encompassed only a minority of the transcribed and flanking regions of *GREM1*, *BMP4* and *BMP2*. We therefore tested for further independent CRC-associated SNPs around these loci (Table S4) by undertaking a pooled analysis of data from 5 CRC GWA studies (UK1, Scotland1, VQ58, CCFR, Australia) and from the UK2 and Scotland2 samples that had been genotyped at 55,000 SNPs with the strongest evidence of association from meta-analysis of UK1 and Scotland1 (Figure S4) [1]. Since each of the 7 sample sets had been genotyped using different, but overlapping, SNP panels, we performed the combined analysis irrespective of the number of studies in which any SNP had been typed. Figure 2 shows the resulting signals of association from single SNP analysis in this discovery phase.

We prioritised SNPs for further assessment in the replication data sets if they passed two thresholds. First, we required SNPs to show association with CRC at *P*<1×10^−4^ under the allelic or Cochran-Armitage tests; this was a less stringent threshold than that used in our previously-reported hypothesis-free GWA studies [1], [2], [3], reflecting the fact that *GREM1*, *BMP4* and *BMP2* were strong candidate susceptibility genes. Four SNPs at *BMP4*, 3 at *BMP2* and 9 at *GREM1* fulfilled this criterion (Figure 2). Second, since our aim was to test for novel, independent disease variants rather than to refine existing signals of association, we required that SNP genotypes were not correlated with each other or with previously identified risk SNPs (r^2^<0.05, D′<0.10). After applying these criteria, one SNP at *BMP4* (rs1957636) and one at *BMP2* (rs4813802) were retained for subsequent analyses.

rs1957636 and rs4813802 were then genotyped in the validation sample sets (Figure S4), comprising 15,075 CRC cases and 13,296 controls from six independent European case-control series (COIN/NBS, UK3, UK4, Scotland3, Cambridge, Helsinki). After combined analysis, significant associations (Table 2) were shown for both rs1957636, *P* = 1.36×10^−9^ (OR = 1.08, 95% CI: 1.06–1.011, *P*_het_ = 0.009, I^2^ = 54%) and rs4813802, *P* = 7.52×10^−11^ (OR = 1.09, 95% CI: 1.06–1.012, *P*_het_ = 0.42, I^2^ = 3%). In case-only analysis, neither SNP showed any evidence of association with age or sex (*P*>0.05, details not shown).

**Table 2:** Summary of individual SNP association analysis for rs4444235, rs1957636, rs961253, and rs4813802.

	Sample series		Case genotypes			Control genotypes					OR
			CC	CT	TT	CC	CT	TT	freqC (cases)	freqC (controls)	
rs4444235	1	UK1	247	441	233	184	470	274	0.508	0.452	1.252
P_overall_ = 1.95×10^−11^	2	Scotland1	220	500	256	195	512	294	0.482	0.451	1.133
OR = 1.091	3	UK2	684	1407	761	639	1322	857	0.487	0.461	1.106
95%CI 1.064–1.119	4	Scotland2	449	1017	540	428	999	630	0.477	0.451	1.112
P_het_ = 0.589, I2 = 0.0%	5	VQ58	410	886	503	603	1312	773	0.474	0.468	1.023
	6	CCFR	298	595	290	227	496	274	0.503	0.476	1.114
	7	Australia	108	208	124	76	233	129	0.482	0.439	1.186
P_discovery_ = 1.61×10^−7^	8	Helsinki	202	459	272	150	405	273	0.462	0.426	1.161
	9	Cambridge	537	1083	618	519	1086	650	0.482	0.471	1.045
P_replication_ = 1.56×10^−5^	10	COIN/NBS	510	1044	593	532	1246	722	0.481	0.462	1.078
	11	UK3	1828	3865	2012	1006	2116	1247	0.488	0.472	1.065
	12	Scotland3	268	554	305	432	1130	628	0.484	0.455	1.121
	13	UK4	127	306	141	210	544	288	0.488	0.463	1.107

			AA	AG	GG	AA	AG	GG	freqA(cases)	freqA(controls)	1.06
rs1957636	1	UK1	171	440	310	151	435	343	0.425	0.397	1.122
P_overall_ = 1.36×10^−9^	2	Scotland1	172	484	320	148	499	354	0.424	0.397	1.118
OR = 1.084	3	UK2	475	1383	1002	457	1348	1044	0.408	0.397	1.046
95%CI 1.056–1.112	4	Scotland2	365	964	677	324	971	761	0.422	0.394	1.125
P_het_ = 0.009, I2 = 54.1%	5	VQ58	302	891	606	445	1303	941	0.416	0.408	1.032
	6	CCFR	206	594	386	142	486	370	0.424	0.386	1.173
	7	Australia	68	220	152	62	208	168	0.405	0.379	1.113
P_discovery_ = 2.79×10^−5^	8	Helsinki	169	462	292	146	397	253	0.433	0.433	1.002
	9	Cambridge	364	1071	788	382	1053	765	0.405	0.413	0.966
P_replication_ = 1.24×10^−5^	10	COIN/NBS	405	1087	690	320	1056	797	0.435	0.390	1.201
	11	UK3	1234	3443	2438	660	2076	1519	0.415	0.399	1.070
	12	Scotland3	223	601	448	326	1160	808	0.412	0.395	1.071
	13	UK4	128	274	182	159	485	400	0.454	0.385	1.329

			AA	AC	CC	AA	AC	CC	freqA(cases)	freqA(controls)	
rs961253	1	UK1	160	418	343	133	423	373	0.401	0.371	1.134
P_overall_ = 4.45×10^−16^	2	Scotland1	151	460	366	127	468	406	0.390	0.361	1.133
OR = 1.117	3	UK2	418	1335	1099	351	1300	1167	0.381	0.355	1.115
95%CI 1.088–1.148	4	Scotland2	300	939	767	265	898	894	0.384	0.347	1.171
P_het_ = 0.379, I2 = 6.8%	5	VQ58	219	771	620	357	1249	1083	0.375	0.365	1.046
	6	CCFR	159	558	469	116	470	410	0.369	0.352	1.076
	7	Australia	63	218	159	58	193	187	0.391	0.353	1.178
P_discovery_ = 1.29×10^−7^	8	Helsinki	115	408	418	72	335	407	0.339	0.294	1.230
	9	Cambridge	287	928	801	275	971	898	0.373	0.355	1.080
P_replication_ = 4.97×10^−10^	10	COIN/NBS	305	1034	843	332	1171	997	0.377	0.367	1.042
	11	UK3	1073	3665	2913	551	1944	1823	0.380	0.353	1.124
	12	Scotland3	177	503	423	260	975	947	0.388	0.343	1.219
	13	UK4	95	271	204	147	474	422	0.404	0.368	1.165

			GG	TG	TT	GG	TG	TT	freqG(cases)	freqG(controls)	
rs4813802	1	UK1	141	425	350	130	398	393	0.386	0.357	1.131
P_overall_ = 7.52×10^−11^	2	Scotland1	147	452	373	151	450	393	0.384	0.378	1.023
OR = 1.093	3	UK2	374	1299	1026	335	1294	1037	0.379	0.368	1.048
95%CI 1.064–1.122	4	Scotland2	359	918	724	277	955	830	0.409	0.366	1.198
P_het_ = 0.416, I2 = 3.1%	5	VQ58	268	832	694	351	1208	1131	0.381	0.355	1.120
	6	CCFR	176	546	460	112	445	436	0.380	0.337	1.206
	7	Australia	53	208	169	53	212	173	0.365	0.363	1.009
P_discovery_ = 2.75×10^−7^	8	Helsinki	131	400	395	88	366	375	0.357	0.327	1.145
	9	Cambridge	280	992	707	284	1039	832	0.392	0.373	1.085
P_replication_ = 3.47×10^−5^	10	COIN/NBS	313	982	818	327	1162	1012	0.381	0.363	1.078
	11	UK3	1037	3582	2969	544	2025	1751	0.373	0.360	1.055
	12	Scotland3	205	523	451	318	1043	878	0.396	0.375	1.091
	13	UK4	92	265	221	102	285	301	0.388	0.355	1.152

Results of allelic test of association in all sample sets are shown.

We used unconditional logistic regression, adjusting for sample series, to test the independence of the two pairs of SNPs at *BMP4* and at *BMP2*. In both cases, each signal remained independent, reflecting the existence of recombination hotspots between the pairs of SNPs at each locus (Figure S5 and Figure S6). For rs4444235 and rs1957636, association *P*-values were respectively 2.09×10^−8^ (I^2^ = 47.7%) and 3.93×10^−10^ (I^2^ = 0%)). For rs961253 and rs4813802, *P*-values were 1.89×10^−15^ (I^2^ = 5%) and 4.65×10^−11^ (I^2^ = 5%)). Thus, all 4 SNPs represented independent signals of association with CRC. Further imputation around *BMP4* and *BMP2* provided no evidence for the alternative possibility that a single variant was tagged by the two SNPs in each region (details not shown).

### Annotation of the regions around rs1957636 and rs4813802

rs1957636 (chr14: 53,629,768) is 136 kb upstream of the transcriptional start site of *BMP4*, 150 kb telomeric to the previously-identified CRC susceptibility SNP, rs4444235 (chr14:53,480,669), which is downstream of *BMP4*. There is a recombination hotspot at chr14:53,510,000 (Figure S5) and LD between rs1957636 and rs4444235 is very weak (r^2^ = 0.004, D′ = 0.073 from UK1). rs1957636 is within a region of LD flanked by SNPs rs431669 (chr14:53,512,418) and rs10150369 (chr14:53,873,515). This region contains no known transcripts, and the nearest gene apart from *BMP4* is *CDKN3* (transcriptional start site, chr14:53,933,476). Using SNAP (http://www.broadinstitute.org/mpg/snap/) to search HapMap3 release 2 and 1000 Genomes Pilot 1, we identified 265 SNPs were in moderate or greater LD (r^2^>0.20) with rs1957636 in Europeans. Of those SNPs, several mapped to sites of potential functional importance in *BMP4* transcription (H3K4Me1, H3K4Me3, DNAseI hypersensitivity, transcription factor ChIP-Seq), as evidenced by the ENCODE regulation tracks (http://genome.ucsc.edu/cgi-bin/hgTrackUi?hgsid=171775907&c=chr14&g=wgEncodeReg) of the UCSC Genome Browser. For example, rs12432287 (r^2^ = 0.60, D′ = 1.00 with rs1957636) and rs728425 (r^2^ = 0.69, D′ = 1.00) lie within a region of apparently high transcriptional regulatory activity at chr14:53,642,340–53,652,937. Another SNP, rs8011813 (r^2^ = 0.822, D′ = 0.811), maps within a similar region at chr14:53, 728, 957–53, 731, 647. Although none of the SNPs in the region around rs1957636 is the location of a reported eQTL (http://eqtl.uchicago.edu/cgi-bin/gbrowse/eqtl/), no studies relating transcription to SNP genotype have yet been undertaken in the colorectum.

rs4813802 maps to chr20:6,647,595, about 49 kb upstream of *BMP2* and 295 kb telomeric of the previously-identified *BMP2* CRC susceptibility SNP, rs961253 (chr20:6,352,281). There is very little LD between these two SNPs (r^2^ = 0.000, D′ = 0.017 from UK1) owing to a recombination hotspot at chr20:6,587,000 (Figure S5). rs4813802 lies within a region of LD flanked by rs727689 (chr20:6,636,405) and rs6117401 (chr20:6,664,097). This region contains 3 spliced ESTs, BX107852, BG822004 and DB094697; none of these has any known functional role or homology to other human or non-human transcripts or genes. The nearest gene to rs4813802 apart from *BMP4* is *FERMT1* (transcriptional start site, chr20:6,052,191). From HapMap3 release 2 and 1000 Genomes Pilot 1, 29 SNPs were found to be in moderate or greater LD (r^2^>0.20) with rs4813802 in Europeans. Of those SNPs, several in the region chr20:6,636,405–6,647,595 mapped to sites of potential functional importance in *BMP2* transcription. None of the SNPs in the area around rs4813802 is the location of a reported eQTL.

### Gene-gene interactions and other SNPs near BMP pathway loci

Using a case-control logistic regression design, we searched for pairwise gene-gene interactions between 5 SNPs associated with CRC risk (rs4444235, rs1957636, rs961253, rs4813802 and rs4779584). Risks were additive and no evidence of epistasis was detected (*P*>0.2 for all SNP pairs).

We also searched for evidence of CRC susceptibility alleles at tagSNPs close to other BMP pathway genes. Using the transcribed regions of flanking genes as boundaries, we identified 4,361 tagSNPs mapping to 37 BMP agonist, antagonist and receptor loci (Table S5). However, we found no statistically significant evidence of associations with disease (*P*>10^−3^ in all cases).

## Discussion

We have identified two new CRC predisposition tagSNPs close to *BMP4* (rs1957636) and *BMP2* (rs4813802). To date, few other loci have been shown at stringent levels of significance to harbour more than one, independent cancer susceptibility variant. One notable exception is the locus proximal to *MYC* on chromosome 8q24.21 that contains multiple regions independently associated with the risk of prostate and other cancers [4]. Low-penetrance cancer predisposition loci are becoming increasingly hard to identify, owing to small effect sizes and/or low risk allele frequencies – and a return to candidate gene-based approaches may become increasingly attractive. It is true that in the past, candidate gene approaches have generally been unsuccessful at identifying cancer risk loci, but it is now possible to make use of information, such as expression quantitative trait locus identification, that increasingly permits a more considered approach.

We have also found good evidence that the original CRC-associated SNP near *GREM1*, rs4779584 [5], tags two independent functional SNPs, represented by association signals at rs16969681 and rs11632715. This finding emphasises that genetic fine-mapping studies are valuable not only for detecting stronger association signals, but also for deconvoluting tagSNP associations that have arisen owing to independent correlation of the tagSNP with more than one functional SNP. The original rs4779584 tagSNP signal could be described as an example of “synthetic association”, a term that has been used to describe a situation in which multiple, sometimes rare, variants underlie a tagSNP signal [6], [7]. Synthetic association can explain some of the apparently missing heritability of complex diseases. Here, we estimate that the 6 SNPs close to the 3 BMP pathway genes contribute approximately 2% of the heritability of CRC, about double that estimated before this study.

Finally, our data provide evidence that *GREM1*, *BMP4* and *BMP2* are the targets of the functional variation in each region. Multiple, independently-acting variants close to these loci contribute to CRC risk. Perhaps unexpectedly, there are no detectable genetic interactions among these variants. If the downstream *SMAD* effectors that function within both the BMP and TGF-beta pathways are included, the components of BMP signalling involved in CRC risk might comprise up to 3 high-penetrance predisposition genes (*SMAD4*, *BMPR1A*, *GREM1*) and 8 low-penetrance variants at *GREM1*, *BMP4*, *BMP2*, *SMAD7* and *LAMA5* (tagged respectively by rs16969681 and rs11632715, rs4444235 and rs1957636, rs961253 and rs4813802, rs4939827, and rs4925386) [1], [2], [3], [5], [8], [9], [10], [11]. Collectively these data emphasise the potential importance of genetic variants in the BMP pathway for CRC predisposition.

## Methods

### Ethics statement

Collection of blood samples and clinico-pathological information from patients and controls was undertaken with informed consent and ethical review board approval in accordance with the tenets of the Declaration of Helsinki.

### Overall strategy

The study had two main components: (i) refinement of existing GWAS signals at the *GREM1*, *BMP4* and *BMP2* loci using a dense genotyping and imputation approach in several thousand cases and controls previously used for GWAS validation; and (ii) a search for new, independent CRC tagSNPs at the same three loci using a less stringent threshold for validation than used previously, combined with multiple validation sample sets.

### Discovery screen data sets

UK1 (CORGI) [1] comprised 922 cases with colorectal neoplasia (47% male) ascertained through the Colorectal Tumour Gene Identification (CoRGI) consortium. All had at least one first-degree relative affected by CRC and one or more of the following phenotypes: CRC at age 75 or less; any colorectal adenoma (CRAd) at age 45 or less; ≥3 colorectal adenomas at age 75 or less; or a large (>1 cm diameter) or aggressive (villous and/or severely dysplastic) adenoma at age 75 or less. The 929 controls (45% males) were spouses or partners unaffected by cancer and without a personal family history (to 2^nd^ degree relative level) of colorectal neoplasia. Known dominant polyposis syndromes, HNPCC/Lynch syndrome or bi-allelic *MYH* mutation carriers were excluded. All cases and controls were of white UK ethnic origin.

Scotland1 (COGS) [1] included 980 CRC cases (51% male; mean age at diagnosis 49.6 years, SD±6.1) and 1,002 cancer-free population controls (51% male; mean age 51.0 years; SD±5.9). Cases were for early age at onset (age ≤55 years). Known dominant polyposis syndromes, HNPCC/Lynch syndrome or bi-allelic *MYH* mutation carriers were excluded. Control subjects were sampled from the Scottish population NHS registers, matched by age (±5 years), gender and area of residence within Scotland.

VQ58 comprised 1,832 CRC cases (1,099 males, mean age of diagnosis 62.5 years; SD±10.9) from the VICTOR [12] and QUASAR2 (www.octo-oxford.org.uk/alltrials/trials/q2.html) trials. There were 2,720 population control genotypes (1,391 males,) from the Wellcome Trust Case-Control Consortium 2 (WTCCC2) 1958 birth cohort (also known as the National Child Development Study), which included all births in England, Wales and Scotland during a single week in 1958 [13].

The Colon Cancer Family Registry (CCFR) data set [14] comprised 1,332 familial CRC cases and 1,084 controls Colon Cancer Family Registry (Colon-CFR) (http://epi.grants.cancer.gov/CFR/about_colon.html). The cases were recently diagnosed CRC cases reported to population complete cancer registries in the USA (Puget Sound, Washington State) who were recruited by the Seattle Familial Colorectal Cancer Registry; in Canada (Ontario) who were recruited by the Ontario Familial Cancer Registry; and in Australia (Melbourne, Victoria) who were recruited by the Australasian Colorectal Cancer Family Study. Controls were population-based and for this analysis were restricted to those without a family history of colorectal cancer.

The Australian study [15] comprised 591 patients treated for CRC at the Royal Melbourne, Western and St Francis Xavier Cabrini Hospitals in Melbourne from 1999 to 2009. The 2,353 controls were derived from Queensland or Melbourne: for the former, the controls came from the Brisbane Twin Nevus Study [16]; for the latter, individuals were participants in the Genes in Myopia study [17]. There was no overlap between the CFR and Australian data sets. Owing to potential residual ethnic heterogeneity within the Melbourne population, for the Australian cohort only we performed an additional screen to minimise heterogeneity after performing principal components analysis (PCA) to remove individuals who clustered with non-CEU individuals (see below). We achieved this by performing PCA on the Australian cases and controls without reference samples of known ancestry. We then paired each case with a control in a 1∶1 ratio based on a maximum separation of 0.050 using the first and second eigenvectors. All unpaired samples were excluded, leaving 441 cases and 441 controls in the study. The genomic inflation factor, λ_GC_, was 1.02 after this filtering.

UK2 (NSCCG) [1] consisted of 2,854 CRC cases (58% male, mean age at diagnosis 59.3 years; SD±8.7) ascertained through two ongoing initiatives at the Institute of Cancer Research/Royal Marsden Hospital NHS Trust (RMHNHST) from 1999 onwards - The National Study of Colorectal Cancer Genetics (NSCCG) and the Royal Marsden Hospital Trust/Institute of Cancer Research Family History and DNA Registry. The 2,822 controls (41% males; mean age 59.8 years; SD±10.8) were the spouses or unrelated friends of patients with malignancies. None had a personal history of malignancy at time of ascertainment. All cases and controls had self-reported European ancestry, and there were no obvious differences in the demography of cases and controls in terms of place of residence within the UK.

Scotland2 (SOCCS) [1] comprised 2,024 CRC cases (61% male; mean age at diagnosis 65.8 years, SD±8.4) and 2,092 population controls (60% males; mean age 67.9 years, SD±9.0) ascertained in Scotland. Cases were taken from an independent, prospective, incident CRC case series and aged <80 years at diagnosis. Control subjects were population controls matched by age (±5 years), gender and area of residence within Scotland.

### Replication data sets

UK3 (NSCCG) [1] comprised 7,912 CRC cases (65% male; mean age at diagnosis 59 years, SD±8.2) and 4,398 controls (40% male; mean age 62 years, SD±11.5) ascertained through NSCCG post-2005.

Scotland3 (SOCCS) [1] comprised 1,145 CRC cases (50% male; mean age at diagnosis 53.2 years, SD±15.4) and 2,203 cancer-free population controls (47% male; mean age 51.8 years, SD±11.5). Controls were recruited as part of the Generation Scotland study.

UK4 (CORGI2BCD) [1] consisted of 621 CRC cases (46% male; mean age at diagnosis 58.3 years; SD±14.1) and 1,121 cancer-free population or spouse controls (45% male; mean age 45.1 years, SD±15.9).

Cambridge/SEARCH consisted of 2,248 CRC cases (56% male; mean age at diagnosis 59.2 years, SD±8.1) and 2,209 controls (42% males; mean age 57.6 years; SD±15.1. Samples were ascertained through the SEARCH (Studies of Epidemiology and Risk Factors in Cancer Heredity, http://www.cancerhelp.org.uk/trials/a-study-looking-at-genetic-causes-of-cancer) study based in Cambridge, UK. Recruitment started in 2000; initial patient contact was though the general practitioner. Control samples were collected post-2003. Eligible individuals were sex- and frequency-matched in five-year age bands to cases.

The COIN samples [18] were 2,151 cases derived from the COIN and COIN-B clinical trials of metastatic CRC. Median age was 63 years. COIN cases were compared against genotypes from 2,501 population controls (1,237 males,) from the WTCCC2 National Blood Service (NBS) cohort (50% male; mean age at diagnosis 53.2 years, SD±15.4).

The Helsinki (FCCPS) study (http://research.med.helsinki.fi/gsb/aaltonen/) comprised 988 cases from a population-based collection centred on south-eastern Finland and 864 population controls from the same collection.

EPICOLON [19] included 1,410 cases matched with the same number of controls collected in a prospective fashion from centres in Spain. Exclusion criteria were Mendelian CRC syndromes and a personal history of inflammatory bowel disease.

In all cases CRC was defined according to the ninth revision of the International Classification of Diseases (ICD) by codes 153–154 and all cases had pathologically proven adenocarcinomas.

### Sample preparation and genotyping

DNA was extracted from samples using conventional methods and quantified using PicoGreen (Invitrogen). The VQ, UK1, Scotland1 and Australia GWA cohorts were genotyped using Illumina Hap300, Hap370, or Hap550 arrays. 1958BC and NBS genotyping was performed as part of the WTCCC2 study on Hap1M arrays. The CCFR samples were genotyped using Illumina Hap1M or Hap1M-Duo arrays. In UK2 and Scotland2, genotyping was conducted using custom Illumina Infinium arrays according to the manufacturer's protocols. Some COIN SNPs were typed on custom Illumina Goldengate arrays. To ensure quality of genotyping, a series of duplicate samples was genotyped, resulting in 99.9% concordant calls in all cases.

Other genotyping was conducted using competitive allele-specific PCR KASPar chemistry (KBiosciences Ltd, Hertfordshire, UK), Taqman (Life Sciences, Carlsbad, California) or MassARRAY (Sequenom Inc., San Diego, USA). All primers, probes and conditions used are available on request. Genotyping quality control was tested using duplicate DNA samples within studies and SNP assays, together with direct sequencing of subsets of samples to confirm genotyping accuracy. For all SNPs, >99% concordant results were obtained.

### Quality control and sample exclusion

We excluded SNPs from analysis if they failed one or more of the following thresholds: GenCall scores <0.25; overall call rates <95%; MAF<0.01; departure from Hardy-Weinberg equilibrium (HWE) in controls at *P*<10^−4^ or in cases at *P*<10^−6^; outlying in terms of signal intensity or X∶Y ratio; discordance between duplicate samples; and, for SNPs with evidence of association, poor clustering on inspection of X∶Y plots.

We excluded individuals from analysis if they failed one or more of the following thresholds: duplication or cryptic relatedness to estimated identity by descent (IBD) >6.25%; overall successfully genotyped SNPs <95%; mismatch between predicted and reported gender; outliers in a plot of heterozygosity *versus* missingness; and evidence of non-white European ancestry by PCA-based analysis in comparison with HapMap samples (http://hapmap.ncbi.nlm.nih.gov/). We excluded 6 duplicate samples using PCA (see below) within the UK samples that had undergone analysis of over 200 SNPs (UK1, Scotland1, UK2, Scotland2, VQ, 1958BC, NBS, COIN). We excluded duplicates from other UK cohorts on the basis of names (or initials where release of names was not possible) and dates of birth. No duplicates were found from the CCFR or Australian sample sets.

To identify individuals who might have non-northern European ancestry, we merged our case and control data from all sample sets with the 60 European (CEU), 60 Nigerian (YRI), and 90 Japanese (JPT) and 90 Han Chinese (CHB) individuals from the International HapMap Project. For each pair of individuals, we calculated genome-wide identity-by-state distances based on markers shared between HapMap2 and our SNP panel, and used these as dissimilarity measures upon which to perform principal components analysis. Principal components analysis was performed using Eigenstrat/SmartPCA using CEU, YRI and HCB HapMap samples as reference. The first two principal components for each individual were plotted and any individual not present in the main CEU cluster (that is, >5% of the PC distance from HapMap CEU cluster centroid) was excluded from subsequent analyses.

We had previously shown the adequacy of the case-control matching and possibility of differential genotyping of cases and controls using Q-Q plots of test statistics in STATA. The inflation factor λ_GC_ was calculated by dividing the mean of the lower 90% of the test statistics by the mean of the lower 90% of the expected values from a χ^2^ distribution with 1 d.f. Deviation of the genotype frequencies in the controls from those expected under HWE was assessed by χ^2^ test (1 d.f.), or Fisher's exact test where an expected cell count was <5.

### SNP selection and genotyping for fine mapping

Regions selected for fine mapping were: chr15:30,733,560–30,802,752; chr14:53,430,973n 53,530,761; and chr20:6,292,730–6,402,661. These corresponded to the haplotype blocks and immediately flanking regions harbouring rs4779584, rs4444235, and rs961253. To define these haplotype blocks and the recombination hotspots harbouring these CRC-associated SNPs, we used Haploview and SequenceLDHot. From dbSNP (build 128), we selected all SNPs between the recombination hotspots flanking the haplotype block. All these SNPs were submitted to Illumina for assay design and those with a design score>0.3 were genotyped on custom arrays in the UK2 and Scotland2 case-control series. In total, we genotyped 81, 42 and 60 SNPs in the 15q13.3, 14q22.2 and 20p12.3 regions respectively. A list of these SNPs is shown in Table S1. Association statistics, using an additive model, were obtained with SNPTEST v2 (www.stats.ox.ac.uk/~marchini/software/gwas/snptest.html). We used genotype data from the 1000 Genomes CEPH (http://www.1000genomes.org/) and HapMap3 CEPH and TSI samples (www.hapmap.org/) and the IMPUTE v2 software (https://mathgen.stats.ox.ac.uk/impute/impute_v2.html) to generate *in silico* genotypes at additional SNPs in all three regions. This imputation resulted in the addition of 74, 113 and 255 markers in the chromosome 15q13.3, 14q22.2 and 20p12.3 regions respectively (for details on imputed and genotyped markers see Table S1). Association meta-analyses only included markers with proper_info scores >0.5, imputed call rates per SNP >0.9 and minor allele frequencies (MAFs) >0.01. Meta-analyses of the two sample sets were carried out with Meta (http://www.stats.ox.ac.uk/~jsliu/meta.html) using the genotype probabilities from IMPUTE v2, where a SNP was not directly typed. To test for the presence of additional independent risk alleles in each region, we carried out logistic regression analysis within each region, both pairwise with the original tagSNP and then in a backwards analysis that included all SNPs with evidence of association in the meta-analysis at P<5×10^−4^.

### Statistical analysis

Association between SNP genotype and disease status was primarily assessed in STATA v10 (http://www.stata.com/) and PLINK v1.07 (http://pngu.mgh.harvard.edu/~purcell/plink/) using allelic and Cochran-Armitage tests (both with 1df) respectively, or by Fisher's exact test where an expected cell count was <5. Genotypic (2df), dominant (1df) and recessive (1df) tests were also performed. The risks associated with each SNP were estimated by allelic, heterozygous and homozygous odds ratios (ORs) using unconditional logistic regression, and associated 95% confidence intervals (CIs) were calculated.

Joint analysis of data generated from multiple phases was conducted using standard methods for combining raw data based on the Mantel-Haenszel method in STATA and PLINK. The reported meta-analysis statistics were derived from analysis of allele frequencies, and joint ORs and 95% CIs were calculated assuming fixed- and random-effects models. Tests of the significance of the pooled effect sizes were calculated using a standard normal distribution. Cochran's Q statistic to test for heterogeneity [20] and the I^2^ statistic [21] to quantify the proportion of the total variation due to heterogeneity were calculated. Large heterogeneity is typically defined as I^2^≥75%. Where significant heterogeneity was identified, results from the random effects model were reported. Alongside, we also performed meta-analysis based on allele dosage (0, 1, 2) and incorporated age and sex as co-variates. Although age and sex are associated with colorectal cancer risk, they were not associated with SNP genotype and did not materially affect the significance of any of the 6 reported associations (details not shown).

We used Haploview software v4.2 (http://www.broadinstitute.org/haploview) to infer the LD structure of the genome in the regions around *GREM1*, *BMP2* and *BMP4*. The combined effects of pairs of loci identified as associated with CRC risk were investigated by multiple logistic regression analysis in PLINK to test for independent effects of each SNP and stratifying by sample series. Evidence for interactive effects between SNPs (epistasis) was assessed by likelihood ratio test assuming an allelic model in PLINK.

The sibling relative risk attributable to a given SNP was calculated using the formula
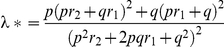
where *p* is the population frequency of the minor allele, *q* = 1−*p*, and *r*_1_ and *r*_2_ are the relative risks (estimated as OR) for heterozygotes and rare homozygotes, relative to common homozygotes [22]. Assuming a multiplicative interaction, the proportion of the familial risk attributable to a SNP was calculated as log(λ^*^)/log(λ_0_), where λ_0_ is the overall familial relative risk estimated from epidemiological studies of CRC, assumed to be 2.2 [23]. UK2/NSCCG2 samples were used for this estimation. The Akaike information criterion was calculated using the swaic command in STATA.

Genome co-ordinates were taken from the NCBI build 36/hg18 (dbSNP b126).

## Supporting Information

Figure S1 Fine mapping around the known CRC risk SNPs close to (a) *BMP4* (14q22) and (b) *BMP2* (20p12).
(DOCX)

Figure S2 Pairwise linkage disequilibrium between rs4779584, rs16969681 and rs11632715 near *GREM1* (upper) and position of recombination hotspot (lower).
(DOCX)

Figure S3 Histone methylation and acetylation marks upstream of *GREM1*.
(DOCX)

Figure S4 Study design for discovery and validation phases.
(DOCX)

Figure S5 Large-scale LD structure in regions around *BMP4* and *BMP2*.
(DOCX)

Figure S6 Locations of recombination hotspots in regions around *BMP4* and *BMP2*.
(DOCX)

Table S1 SNPs genotyped directly or predicted by imputation in the fine mapping of the regions around rs4779584, rs4444235, and rs961253 in UK2 and Scotland2.
(DOCX)

Table S2 Haplotype risk analysis at rs16969681 and rs4779584.
(DOCX)

Table S3 Logistic regression model analysis of CRC risk and genotypes at rs4779584, rs16969681, and rs11632715.
(DOCX)

Table S4 TagSNPs around *GREM1*, *BMP4*, and *BMP2* analysed for new associations.
(DOCX)

Table S5 Additional BMP pathway genes around which tagSNP associations with CRC were analysed.
(DOCX)

Text S1 Consortium co-authors.
(DOCX)

## References

[1] Houlston RS, Cheadle J, Dobbins SE, Tenesa A, Jones AM (2010). Meta-analysis of three genome-wide association studies identifies susceptibility loci for colorectal cancer at 1q41, 3q26.2, 12q13.13 and 20q13.33.. Nat Genet.

[2] Tomlinson I, Webb E, Carvajal-Carmona L, Broderick P, Kemp Z (2007). A genome-wide association scan of tag SNPs identifies a susceptibility variant for colorectal cancer at 8q24.21.. Nat Genet.

[3] Houlston RS, Webb E, Broderick P, Pittman AM, Di Bernardo MC (2008). Meta-analysis of genome-wide association data identifies four new susceptibility loci for colorectal cancer.. Nat Genet.

[4] Al Olama AA, Kote-Jarai Z, Giles GG, Guy M, Morrison J (2009). Multiple loci on 8q24 associated with prostate cancer susceptibility.. Nat Genet.

[5] Jaeger E, Webb E, Howarth K, Carvajal-Carmona L, Rowan A (2008). Common genetic variants at the CRAC1 (HMPS) locus on chromosome 15q13.3 influence colorectal cancer risk.. Nat Genet.

[6] Dickson S, Wang K, Krantz I, Hakonarson H, Goldstein D (2010). Rare variants create synthetic genome-wide associations.. PLoS Biol.

[7] Wray N, Purcell SM, Visscher PM (2011). Synthetic associations created by rare variants do not explain most GWAS results.. PLoS Biol.

[8] Broderick P, Carvajal-Carmona L, Pittman AM, Webb E, Howarth K (2007). A genome-wide association study shows that common alleles of SMAD7 influence colorectal cancer risk.. Nat Genet.

[9] Howe JR, Bair JL, Sayed MG, Anderson ME, Mitros FA (2001). Germline mutations of the gene encoding bone morphogenetic protein receptor 1A in juvenile polyposis.. Nat Genet.

[10] Howe JR, Roth S, Ringold JC, Summers RW, Jarvinen HJ (1998). Mutations in the SMAD4/DPC4 gene in juvenile polyposis.. Science.

[11] Jaeger EE, Woodford-Richens KL, Lockett M, Rowan AJ, Sawyer EJ (2003). An ancestral Ashkenazi haplotype at the HMPS/CRAC1 locus on 15q13-q14 is associated with hereditary mixed polyposis syndrome.. Am J Hum Genet.

[12] Midgley RS, McConkey CC, Johnstone EC, Dunn JA, Smith JL (2010). Phase III randomized trial assessing rofecoxib in the adjuvant setting of colorectal cancer: final results of the VICTOR trial.. J Clin Oncol.

[13] Power C, Jefferis BJ, Manor O, Hertzman C (2006). The influence of birth weight and socioeconomic position on cognitive development: Does the early home and learning environment modify their effects?. J Pediatr.

[14] Newcomb PA, Baron J, Cotterchio M, Gallinger S, Grove J (2007). Colon Cancer Family Registry: an international resource for studies of the genetic epidemiology of colon cancer.. Cancer Epidemiol Biomarkers Prev.

[15] Tie J, Gibbs P, Lipton L, Christie M, Jorissen RN (2010). Optimizing targeted therapeutic development: Analysis of a colorectal cancer patient population with the BRAF(V600E) mutation.. Int J Cancer.

[16] Duffy DL, Iles MM, Glass D, Zhu G, Barrett JH (2010). IRF4 variants have age-specific effects on nevus count and predispose to melanoma.. Am J Hum Genet.

[17] Baird PN, Schache M, Dirani M (2010). The GEnes in Myopia (GEM) study in understanding the aetiology of refractive errors.. Prog Retin Eye Res.

[18] Adams R, Meade A, Wasan H, Griffiths G, Maughan T (2008). Cetuximab therapy in first-line metastatic colorectal cancer and intermittent palliative chemotherapy: review of the COIN trial.. Expert Rev Anticancer Ther.

[19] Abuli A, Bessa X, Gonzalez JR, Ruiz-Ponte C, Caceres A (2010). Susceptibility genetic variants associated with colorectal cancer risk correlate with cancer phenotype.. Gastroenterology.

[20] Petitti DB (1994). Coronary heart disease and estrogen replacement therapy. Can compliance bias explain the results of observational studies?. Ann Epidemiol.

[21] Higgins JP, Thompson SG (2002). Quantifying heterogeneity in a meta-analysis.. Stat Med.

[22] Houlston RS, Ford D (1996). Genetics of coeliac disease.. QJM.

[23] Johns LE, Houlston RS (2001). A systematic review and meta-analysis of familial colorectal cancer risk.. Am J Gastroenterol.

