# Supplementary material for: Multiple Common Susceptibility Variants near BMP Pathway Loci GREM1, BMP4, and BMP2 Explain Part of the Missing Heritability of Colorectal Cancer
Source: PLoS Genet. 2011 Jun 2;7(6):e1002105. doi: 10.1371/journal.pgen.1002105 (PMC3107194; doi:10.1371/journal.pgen.1002105)
Supplement: Figure S1 — Fine mapping around the known CRC risk SNPs close to (a) BMP4 (14q22) and (b) BMP2 (20p12). (DOCX) [file pgen.1002105.s001.docx]

*Supplemental Figure 1. Fine mapping around the known CRC risk SNPs close to (a) BMP4 (14q22) and (b) BMP2 (20p12).*

Results for meta-analysis of UK2 and Scotland2 are shown. Both significance of association (-log_10_(P)) and effect size (β) are presented. The original CRC-associated tagSNPs are shown in blue. Near *BMP4,* the strongest signal is at the original tagSNP, rs4444235. rs961253, the original tagSNP near *BMP2,* lies in a region of about 40kb in which SNPs are in strong LD and thus show very similar signals of association.

(a)

*
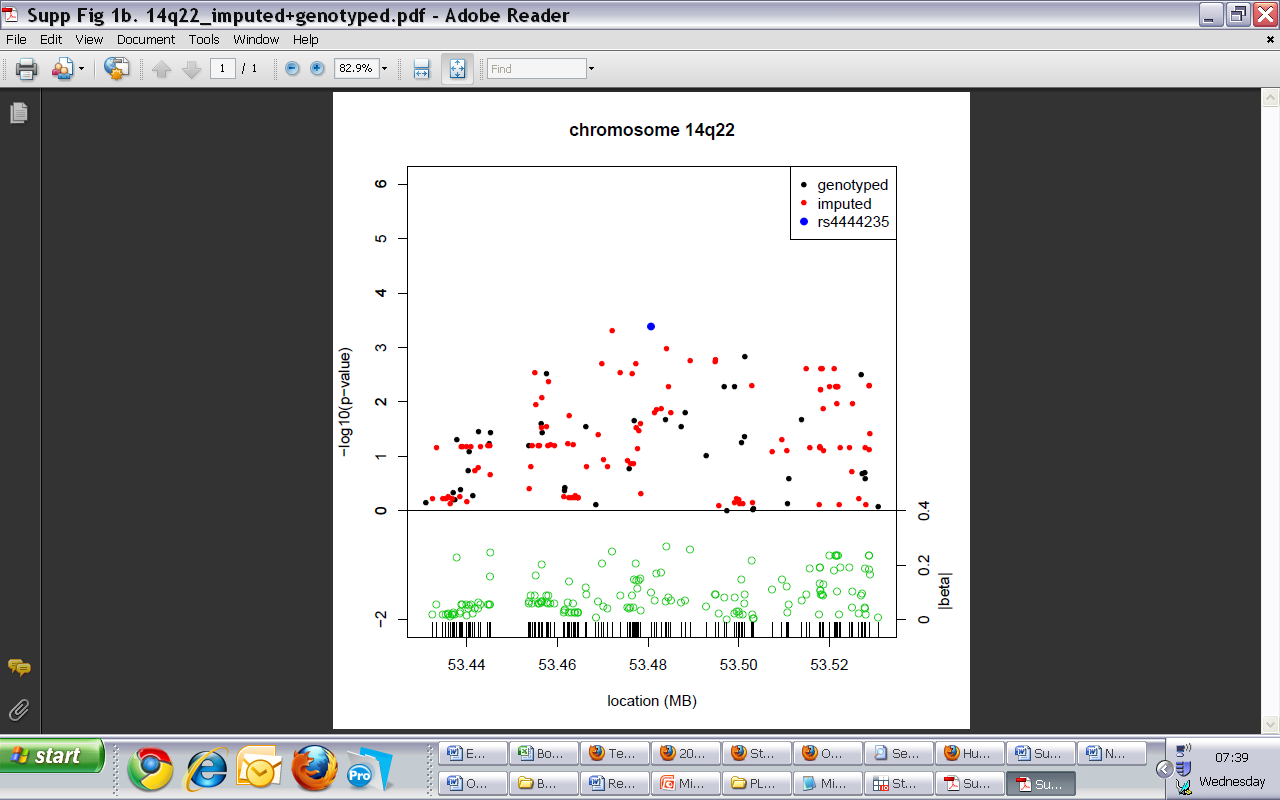
*

(b)

*
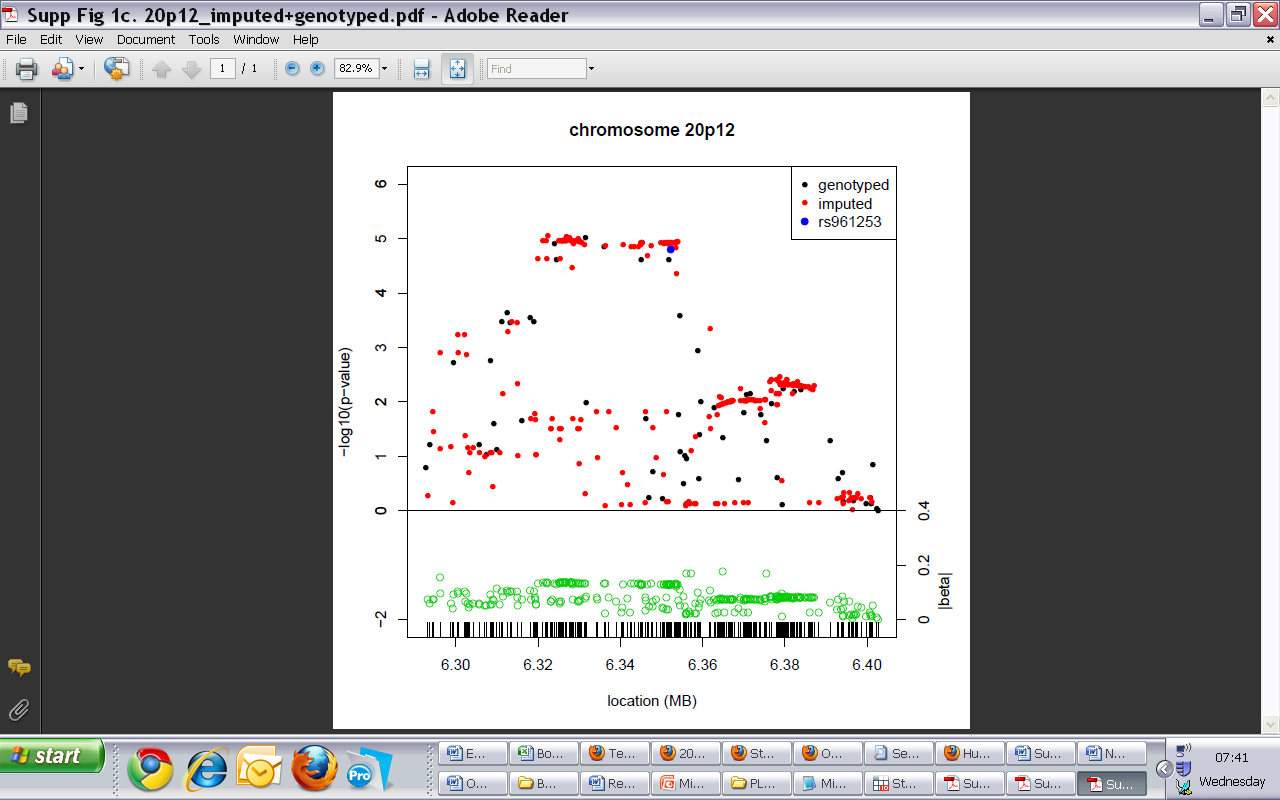
*
