# Supplementary material for: Multiple Common Susceptibility Variants near BMP Pathway Loci GREM1, BMP4, and BMP2 Explain Part of the Missing Heritability of Colorectal Cancer
Source: PLoS Genet. 2011 Jun 2;7(6):e1002105. doi: 10.1371/journal.pgen.1002105 (PMC3107194; doi:10.1371/journal.pgen.1002105)

*Supplemental Figure 2. Pairwise linkage disequilibrium between rs4779584, rs16969681 and rs11632715 near GREM1 (upper) and position of recombination hotspot (lower).*

r^2^ is shown left and D’, right. Data are from HapMap 2 CEU. Standard Haploview colour schemes are used (http://www.haploview.org/).


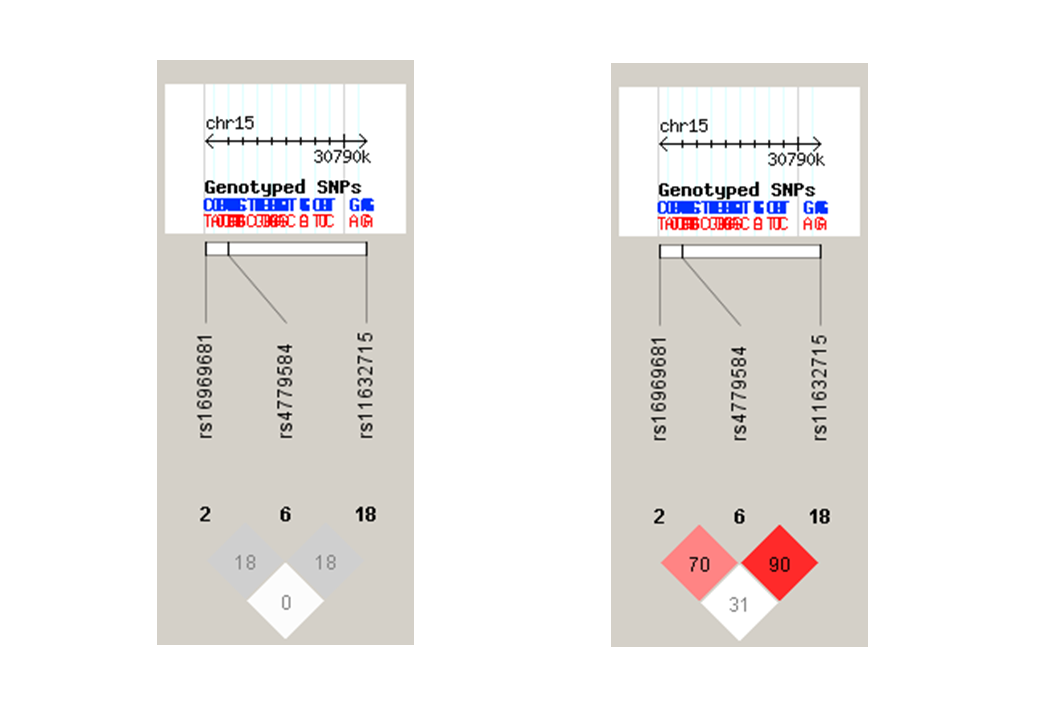


The plot from SNAP shows location of recombination hotspot (peak of blue line, right-hand Y-axis) between rs16969681 and rs11632715. Left-hand Y-axis shows single-SNP association –log_10_(*P*) from logistic regression analysis based on samples genotyped for all 3 SNPs. X-axis shows physical distance.


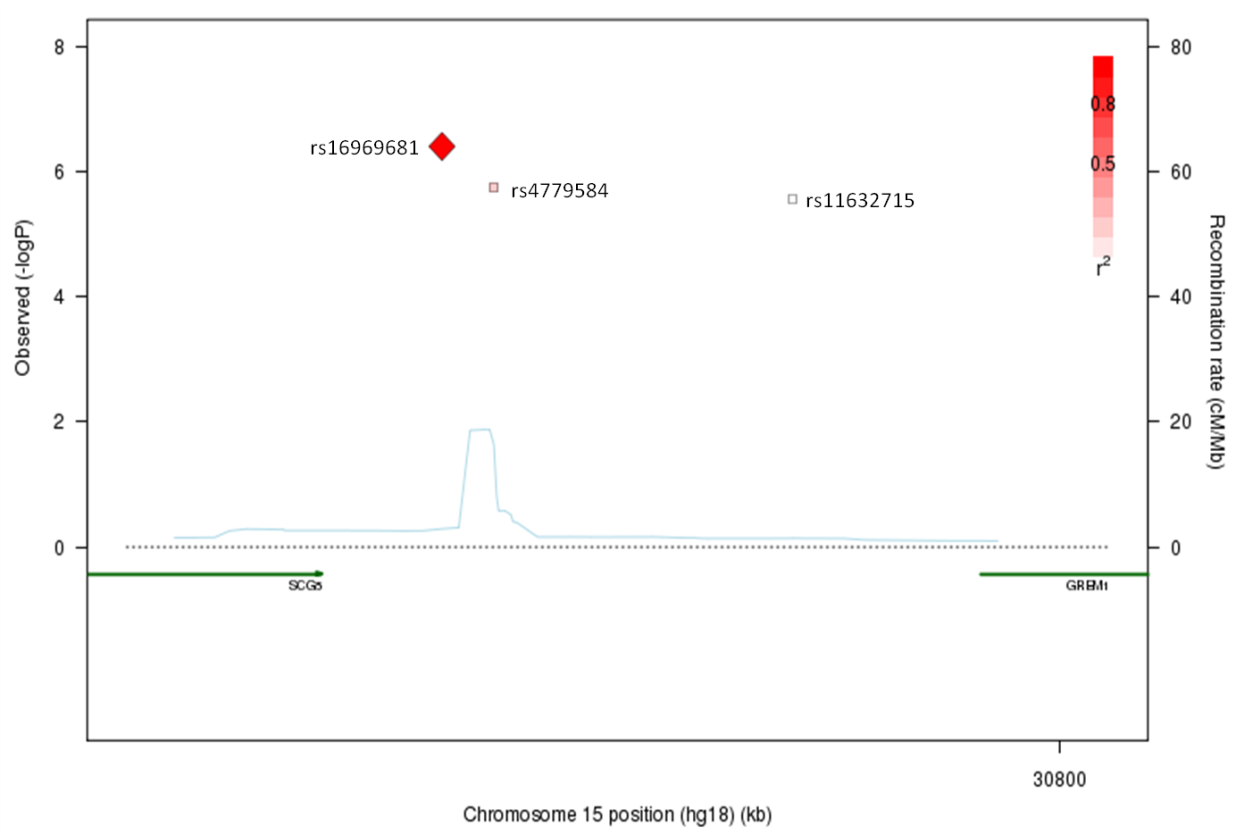

Supplement: Figure S2 — Pairwise linkage disequilibrium between rs4779584, rs16969681 and rs11632715 near GREM1 (upper) and position of recombination hotspot (lower). (DOCX) [file pgen.1002105.s002.docx]
