# Supplementary material for: Multiple Common Susceptibility Variants near BMP Pathway Loci GREM1, BMP4, and BMP2 Explain Part of the Missing Heritability of Colorectal Cancer
Source: PLoS Genet. 2011 Jun 2;7(6):e1002105. doi: 10.1371/journal.pgen.1002105 (PMC3107194; doi:10.1371/journal.pgen.1002105)

*Supplemental Figure 3. Histone methylation and acetylation marks upstream of GREM1.*

To search for possible regulatory elements tagged by SNPs in the 15q13.3 region, we carried out chromatin immunoprecipitation (ChIP) experiments to look for histone modifications between chr15:30,779,300 and the transcriptional start site of *GREM1.* Histone H4 acetylation; and histone H3 methylation at lysine 4 mark active chromatin and intergenic regions showing enrichment for such modifications may contain enhancer elements. We selected three CRC cell lines that express *GREM1* – LS180, C99 and HCC56 – and a control non-expressing cell line, SW48 and carried out ChIP on native chromatin using PCR assays spaced at ~1kb intervals.

ChIP experiments were carried out as previously described (9). Briefly, ~5 x10^7^ cells were collected from four colorectal cancer cell lines (LS180, C99, HCC56 and SW48) and washed in PBS. Nuclei were purified through a sucrose cushion and incubated with MNase to obtain fragments of one to five nucleosomes in length. Approximately 20 µg of native chromatin was incubated with 5-10 µg of antibody overnight at 4°C. The following antibodies were used: H3AcK1, 5, 9 and K14 (06-866) and H3K4me2 (07-030; Millipore). The antibody chromatin complexes were captured with Protein A magnetic beads (Invitrogen). After washing and elution DNA was extracted from the input chromatin, and bound fractions. Q-PCR analysis was carried out using Fast SYBR Green Master Mix using a 7900HT Fast Real-time PCR system (Applied Biosystems). Results were normalized to the Rhodopsin gene (a non-expressed control) and the relative enrichment of the bound fraction compared to the input was calculated using the ΔΔCt method. Primer and probe sequences are available on request.

Plots show the enrichment of antibody bound chromatin relative to input chromatin plotted against the chromosome 15 co-ordinates for the average of the three *GREM 1* expressing lines compared with the non-expressing line. The positions of selected SNPs are shown. Peaks were found, as expected, at the *GREM1* transcriptional start site, but there was a double peak of both acetylation and lysine 4 methylation centred on the region containing rs16969681. This peak was not seen in the control line. rs16969681 is therefore an excellent candidate functional SNP at the *GREM1* locus. The importance of the double-peak centred on rs1881538 is unclear given that it may also be present in the control. We tested rs1881538 in unconditional logistic regression analysis with rs16969681 and rs4779584 to determine whether it might be an association signal tagged by rs4779584 and independent of rs16969681. However, rs4779584 consistently captured more of the variation in disease risk than rs1881538 whether or not rs16969681 was included in the regression model. rs11632715 was not associated with transcription control elements, whether from the ENCODE project or from our own data. It was, however, in LD with several SNPs in potential functionally important regions, including strong association with rs1919364 at the start of the *GREM1* promoter CpG island.


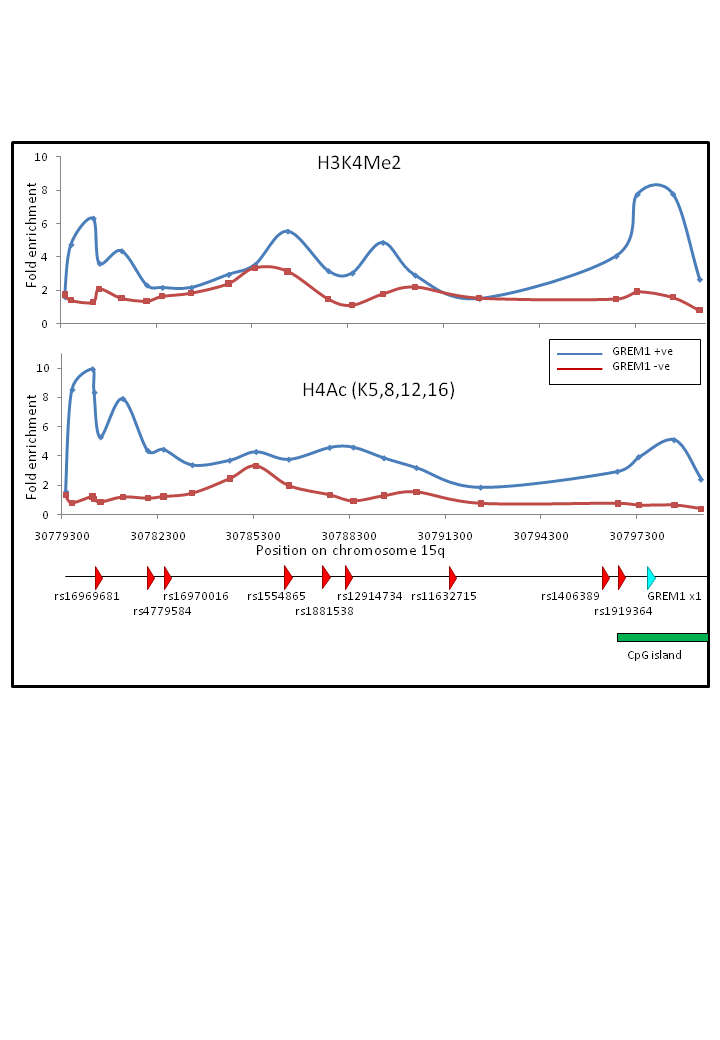

Supplement: Figure S3 — Histone methylation and acetylation marks upstream of GREM1. (DOCX) [file pgen.1002105.s003.docx]
