# Supplementary material for: Multiple Common Susceptibility Variants near BMP Pathway Loci GREM1, BMP4, and BMP2 Explain Part of the Missing Heritability of Colorectal Cancer
Source: PLoS Genet. 2011 Jun 2;7(6):e1002105. doi: 10.1371/journal.pgen.1002105 (PMC3107194; doi:10.1371/journal.pgen.1002105)

*Supplemental Figure 4. Study design for discovery and validation phases.*

The proprietary Illumina arrays used for the 5 GWA studies are shown.


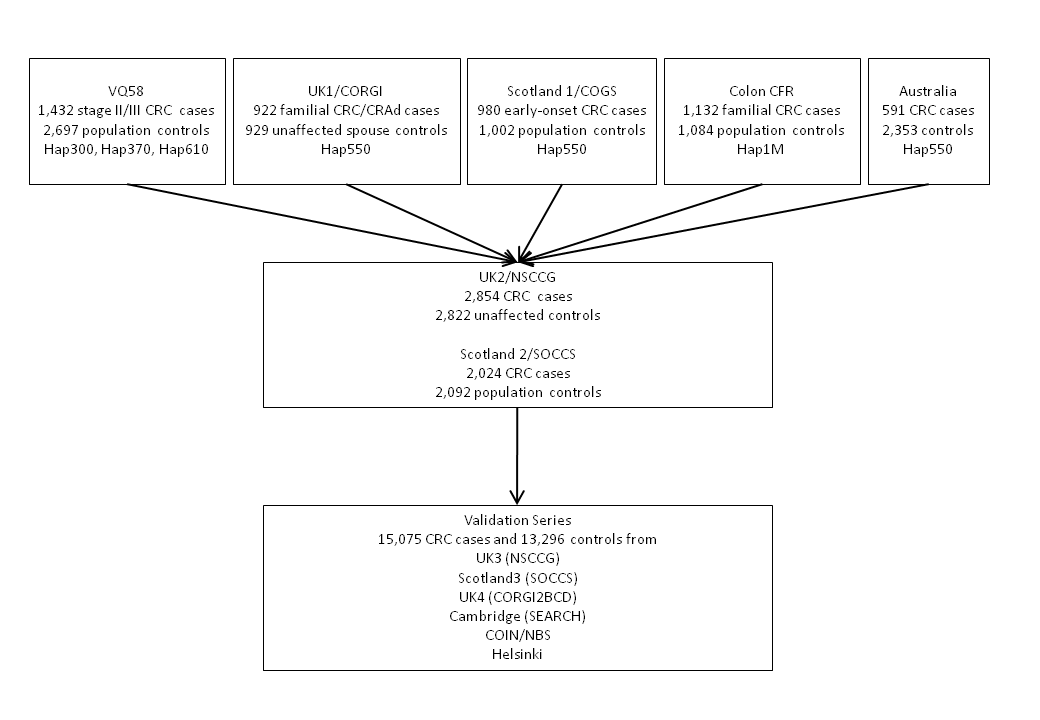

Supplement: Figure S4 — Study design for discovery and validation phases. (DOCX) [file pgen.1002105.s004.docx]
