# Supplementary material for: Multiple Common Susceptibility Variants near BMP Pathway Loci GREM1, BMP4, and BMP2 Explain Part of the Missing Heritability of Colorectal Cancer
Source: PLoS Genet. 2011 Jun 2;7(6):e1002105. doi: 10.1371/journal.pgen.1002105 (PMC3107194; doi:10.1371/journal.pgen.1002105)

*Supplemental Figure 5. Large-scale LD structure in regions around BMP4 and BMP2.*

For each of *BMP2* and *BMP4,* the upper panel shows r^2^ and the middle panel, D’ (from SNAPData are from HapMap2 CEU samples in Haploview). In all cases, X-axes show physical distance. The original tagSNP is shown by a black star and the new signal by a red star.


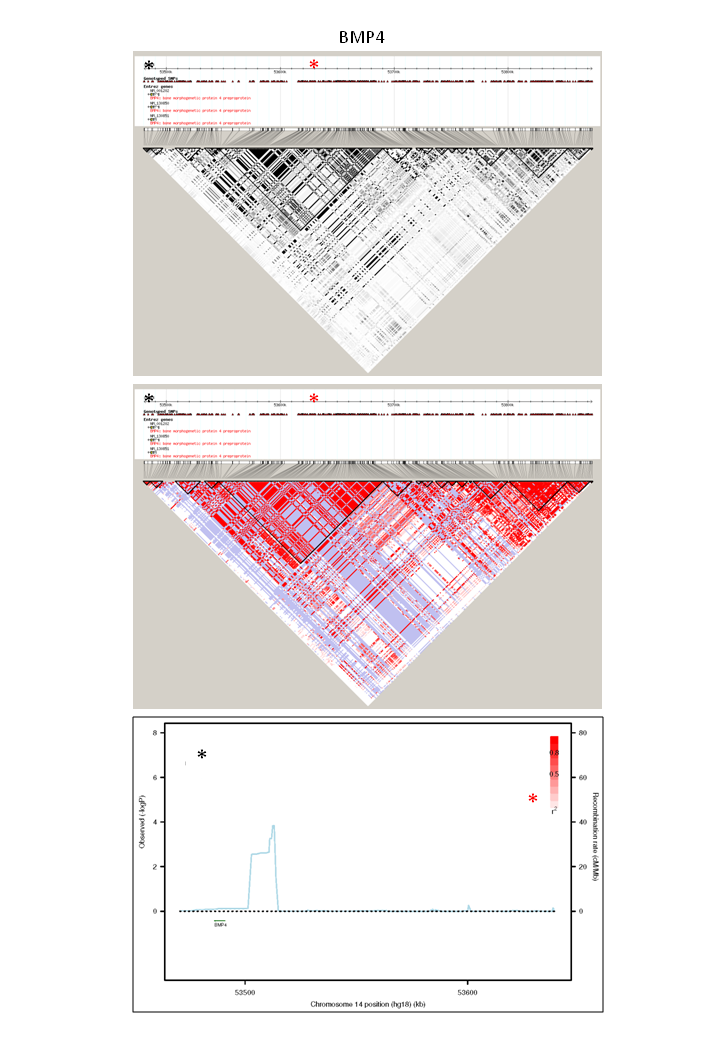


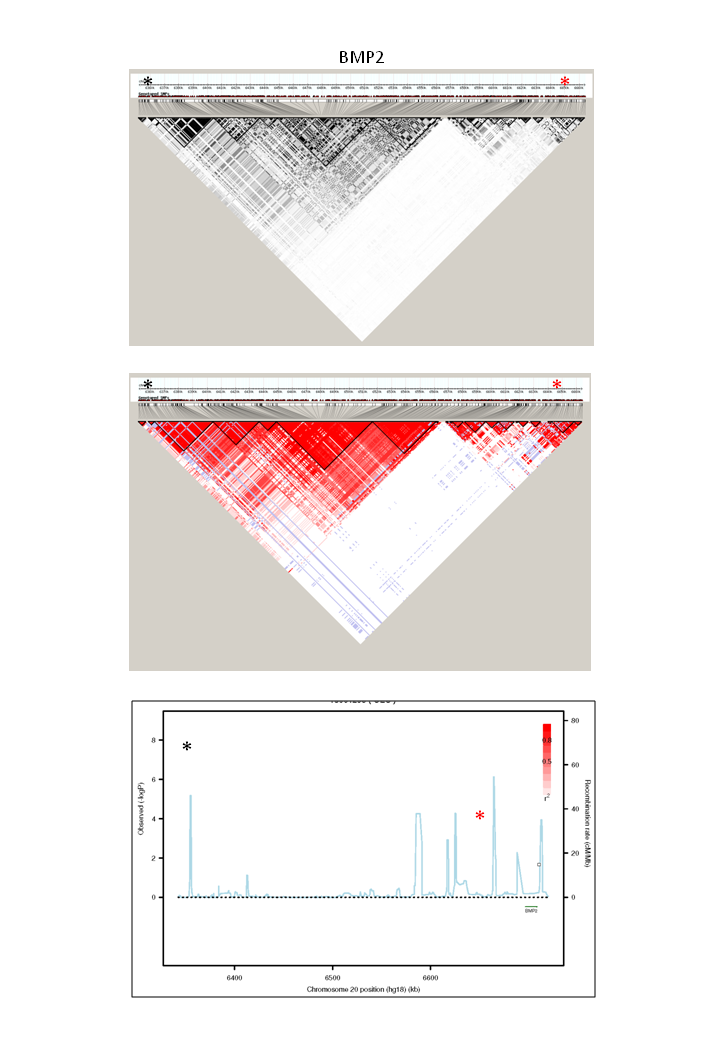

Supplement: Figure S5 — Large-scale LD structure in regions around BMP4 and BMP2. (DOCX) [file pgen.1002105.s005.docx]
