# Supplementary material for: Multiple Common Susceptibility Variants near BMP Pathway Loci GREM1, BMP4, and BMP2 Explain Part of the Missing Heritability of Colorectal Cancer
Source: PLoS Genet. 2011 Jun 2;7(6):e1002105. doi: 10.1371/journal.pgen.1002105 (PMC3107194; doi:10.1371/journal.pgen.1002105)

*Supplemental Figure 6. Locations of recombination hotspots in regions around BMP4 and BMP2.* The plots, from SNAP, show location of recombination hotspots (peaks of blue line, right-hand Y-axis) for the regions between the original tag SNPs near *BMP4* (rs4444235) and *BMP2* (rs961253) and the new signals (rs1957636 and rs4813802 respectively). X-axes show physical distance. Note the presence of hotspots between the locations of the pairs of SNPs.


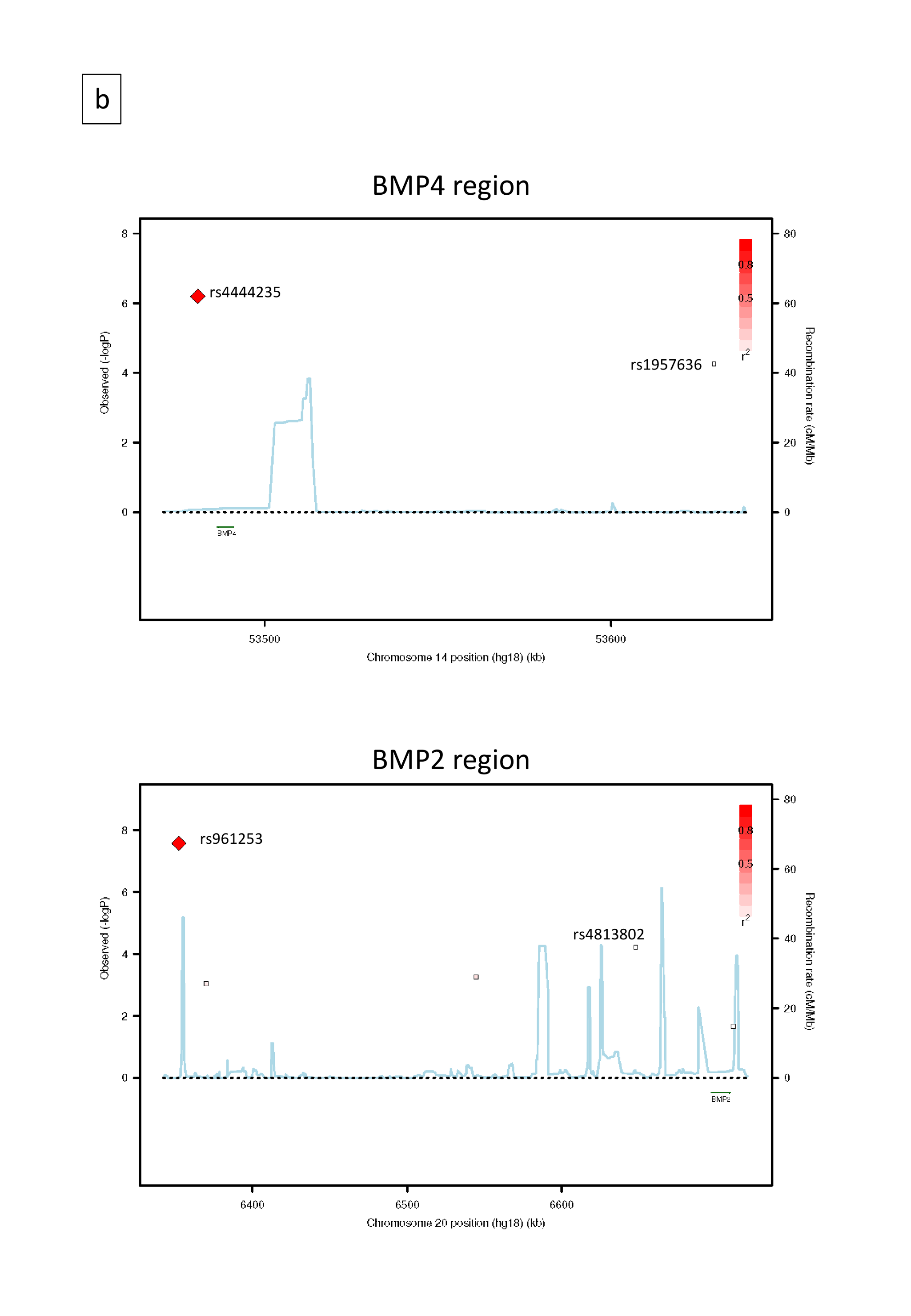

Supplement: Figure S6 — Locations of recombination hotspots in regions around BMP4 and BMP2. (DOCX) [file pgen.1002105.s006.docx]
