# Supplementary material for: Multiple Common Susceptibility Variants near BMP Pathway Loci GREM1, BMP4, and BMP2 Explain Part of the Missing Heritability of Colorectal Cancer
Source: PLoS Genet. 2011 Jun 2;7(6):e1002105. doi: 10.1371/journal.pgen.1002105 (PMC3107194; doi:10.1371/journal.pgen.1002105)
Supplement: Table S1 — SNPs genotyped directly or predicted by imputation in the fine mapping of the regions around rs4779584, rs4444235, and rs961253 in UK2 and Scotland2. (DOCX) [file pgen.1002105.s007.docx]

*Supplemental Table 1. SNPs genotyped directly or predicted by imputation in the fine mapping of the regions around rs4779584, rs4444235 and rs961253 in UK2 and Scotland2.*

We undertook fine mapping of the genomic regions containing CRC-associated tag SNPs close to *GREM1* (15q13.3)*, BMP4* (14q22.2) and *BMP2* (20p12.3)*.* We identified all SNPs in the haplotype blocks and immediately flanking regions for rs4779584, rs4444235 and rs961253 from dbSNP (see Methods) and selected those with minor allele frequency >5%. After excluding SNPs that failed genotyping assay design or that fell below standard quality control (QC) thresholds, a total of 74, 113 and 255 SNPs in each region were successfully genotyped in the CRC cases and controls from the UK2 and Scotland2 sample sets. We then used 1000 Genomes and HapMap 3 reference genotypes to impute untyped SNPs mapping to these regions (1).

**Region Location SNP ID Imputed or Genotyped**

15q13.3 30,733,560 rs12903437 Genotyped

15q13.3 30,734,914 rs11638385 Genotyped

15q13.3 30,734,931 rs11638089 Genotyped

15q13.3 30,735,171 rs7171657 Genotyped

15q13.3 30,735,386 rs12593101 Genotyped

15q13.3 30,737,358 rs55683307 Imputed

15q13.3 30,739,485 rs8023613 Genotyped

15q13.3 30,740,356 rs4238560 Genotyped

15q13.3 30,742,387 rs11071887 Genotyped

15q13.3 30,746,003 rs1406387 Genotyped

15q13.3 30,747,917 rs10083612 Genotyped

15q13.3 30,749,197 rs17228564 Genotyped

15q13.3 30,749,532 rs17228571 Imputed

15q13.3 30,749,742 rs11855680 Imputed

15q13.3 30,749,934 rs8041254 Genotyped

15q13.3 30,750,051 rs8037112 Imputed

15q13.3 30,750,497 rs16963973 Genotyped

15q13.3 30,751,048 rs16964074 Genotyped

15q13.3 30,752,649 rs12909871 Genotyped

15q13.3 30,752,922 rs11634086 Genotyped

15q13.3 30,753,521 rs11635362 Genotyped

15q13.3 30,755,075 rs62001857 Imputed

15q13.3 30,755,131 rs7161975 Genotyped

15q13.3 30,755,270 rs10775188 Genotyped

15q13.3 30,756,123 rs12148394 Genotyped

15q13.3 30,756,146 15-30756146 Imputed

15q13.3 30,756,215 rs7170561 Genotyped

15q13.3 30,756,749 rs10519737 Imputed

15q13.3 30,757,201 rs28688414 Imputed

15q13.3 30,757,349 rs7177176 Genotyped

15q13.3 30,757,435 rs6494576 Imputed

15q13.3 30,757,448 rs11633548 Genotyped

15q13.3 30,757,493 rs16965041 Imputed

15q13.3 30,757,554 rs6494577 Imputed

15q13.3 30,757,842 rs1881536 Genotyped

15q13.3 30,758,358 rs71462819 Imputed

15q13.3 30,758,440 rs11071897 Genotyped

15q13.3 30,758,464 rs34278216 Imputed

15q13.3 30,758,577 rs16965254 Imputed

15q13.3 30,758,627 rs35415313 Imputed

15q13.3 30,758,727 rs12102176 Imputed

15q13.3 30,759,050 rs3817592 Genotyped

15q13.3 30,759,226 rs7177843 Genotyped

15q13.3 30,759,546 15-30759546 Imputed

15q13.3 30,760,107 15-30760107 Imputed

15q13.3 30,760,231 rs12442397 Genotyped

15q13.3 30,760,289 rs12438604 Genotyped

15q13.3 30,760,617 15-30760617 Imputed

15q13.3 30,762,805 rs35703183 Imputed

15q13.3 30,762,980 rs12441136 Genotyped

15q13.3 30,763,009 rs12441140 Genotyped

15q13.3 30,763,347 rs17816224 Imputed

15q13.3 30,763,475 15-30763475 Imputed

15q13.3 30,764,393 rs7165737 Genotyped

15q13.3 30,764,688 15-30764688 Imputed

15q13.3 30,764,833 rs11634570 Imputed

15q13.3 30,765,042 rs56338436 Imputed

15q13.3 30,766,061 rs7403093 Imputed

15q13.3 30,766,905 rs16966853 Genotyped

15q13.3 30,767,015 rs11638903 Genotyped

15q13.3 30,767,310 rs8043234 Genotyped

15q13.3 30,768,135 rs28417809 Imputed

15q13.3 30,768,217 rs4780033 Imputed

15q13.3 30,768,377 rs8039575 Imputed

15q13.3 30,768,468 15-30768468 Imputed

15q13.3 30,768,526 15-30768526 Imputed

15q13.3 30,768,620 rs7178316 Genotyped

15q13.3 30,768,935 rs6494587 Genotyped

15q13.3 30,769,270 rs8024160 Imputed

15q13.3 30,771,488 rs6494593 Imputed

15q13.3 30,772,597 rs17228585 Genotyped

15q13.3 30,772,770 rs12592208 Genotyped

15q13.3 30,773,082 rs28494912 Imputed

15q13.3 30,773,246 rs1997317 Genotyped

15q13.3 30,773,279 rs1997316 Genotyped

15q13.3 30,773,663 rs17228592 Genotyped

15q13.3 30,773,727 rs68050511 Imputed

15q13.3 30,774,389 rs16968154 Imputed

15q13.3 30,774,508 15-30774508 Imputed

15q13.3 30,774,799 15-30774799 Imputed

15q13.3 30,775,388 rs1406388 Genotyped

15q13.3 30,775,430 rs7494781 Genotyped

15q13.3 30,779,005 rs16969344 Genotyped

15q13.3 30,779,950 rs28399071 Imputed

15q13.3 30,780,036 rs8035130 Genotyped

15q13.3 30,780,128 rs12708491 Imputed

15q13.3 30,780,403 rs16969681 Genotyped

15q13.3 30,780,585 rs28436737 Imputed

15q13.3 30,780,817 rs62002603 Imputed

15q13.3 30,780,864 rs28590240 Imputed

15q13.3 30,780,937 rs16969816 Genotyped

15q13.3 30,781,152 rs28630996 Imputed

15q13.3 30,781,293 rs16969862 Genotyped

15q13.3 30,781,348 rs12591992 Genotyped

15q13.3 30,781,704 rs12592056 Genotyped

15q13.3 30,781,917 rs12594722 Genotyped

15q13.3 30,782,069 rs4779585 Genotyped

15q13.3 30,782,135 rs9888701 Genotyped

15q13.3 30,782,465 rs11071915 Imputed

15q13.3 30,782,590 rs16970016 Genotyped

15q13.3 30,782,646 rs9888705 Imputed

15q13.3 30,782,760 rs6494598 Genotyped

15q13.3 30,782,792 rs6494599 Imputed

15q13.3 30,783,056 rs1124774 Imputed

15q13.3 30,783,368 rs28663519 Imputed

15q13.3 30,783,431 rs17816236 Genotyped

15q13.3 30,783,506 rs55659128 Imputed

15q13.3 30,783,535 rs28650777 Imputed

15q13.3 30,784,008 rs12901827 Genotyped

15q13.3 30,784,223 rs12906413 Imputed

15q13.3 30,784,442 rs3861195 Genotyped

15q13.3 30,784,467 rs12902616 Genotyped

15q13.3 30,784,874 rs11853552 Genotyped

15q13.3 30,784,959 rs11857190 Imputed

15q13.3 30,785,128 rs11857997 Genotyped

15q13.3 30,786,603 rs8031842 Imputed

15q13.3 30,786,715 rs6494601 Imputed

15q13.3 30,786,888 15-30786888 Imputed

15q13.3 30,787,098 rs1554865 Genotyped

15q13.3 30,788,001 rs1881538 Genotyped

15q13.3 30,788,556 rs12914734 Genotyped

15q13.3 30,788,858 rs7182252 Genotyped

15q13.3 30,789,026 rs58658771 Imputed

15q13.3 30,789,764 rs11071922 Imputed

15q13.3 30,789,986 rs34054489 Imputed

15q13.3 30,790,000 15-30790000 Imputed

15q13.3 30,790,002 15-30790002 Imputed

15q13.3 30,790,143 15-30790143 Imputed

15q13.3 30,790,156 15-30790156 Imputed

15q13.3 30,790,171 15-30790171 Imputed

15q13.3 30,790,230 15-30790230 Imputed

15q13.3 30,791,354 rs11638007 Genotyped

15q13.3 30,791,539 rs11632715 Genotyped

15q13.3 30,793,167 rs1534594 Genotyped

15q13.3 30,793,696 rs12591802 Genotyped

15q13.3 30,793,707 rs12594148 Imputed

15q13.3 30,793,800 rs12592288 Genotyped

15q13.3 30,793,902 rs12592312 Imputed

15q13.3 30,794,643 rs28473724 Imputed

15q13.3 30,795,651 rs11633862 Genotyped

15q13.3 30,796,770 rs1406389 Genotyped

15q13.3 30,796,866 rs1919364 Imputed

15q13.3 30,797,704 rs2293582 Imputed

15q13.3 30,798,028 rs2293581 Genotyped

15q13.3 30,798,419 rs7168877 Imputed

15q13.3 30,798,933 rs9806137 Genotyped

15q13.3 30,798,989 15-30798989 Imputed

15q13.3 30,799,143 rs8034965 Genotyped

15q13.3 30,799,524 rs11635984 Genotyped

15q13.3 30,799,794 rs73376930 Imputed

15q13.3 30,801,062 rs4779586 Imputed

15q13.3 30,801,587 rs7167214 Genotyped

15q13.3 30,802,498 rs1528734 Genotyped

15q13.3 30,802,694 rs7497354 Genotyped

15q13.3 30,802,752 rs9920024 Genotyped

**Region Location SNP ID Imputed or Genotyped**

14q22.2 53,430,973 rs4898815 Genotyped

14q22.2 53,432,423 rs4898816 Imputed

14q22.2 53,433,295 rs12881924 Imputed

14q22.2 53,434,718 rs56132091 Imputed

14q22.2 53,435,381 rs4901464 Imputed

14q22.2 53,435,871 rs7140284 Imputed

14q22.2 53,435,915 rs7140441 Imputed

14q22.2 53,436,318 rs4901465 Imputed

14q22.2 53,436,788 rs10144601 Imputed

14q22.2 53,436,952 rs4898817 Genotyped

14q22.2 53,437,038 rs1957847 Genotyped

14q22.2 53,437,350 rs12435627 Genotyped

14q22.2 53,437,781 rs2358427 Genotyped

14q22.2 53,438,458 rs12433303 Imputed

14q22.2 53,438,622 rs2181733 Genotyped

14q22.2 53,438,796 rs2147102 Imputed

14q22.2 53,439,049 rs1951864 Imputed

14q22.2 53,439,923 rs2181734 Imputed

14q22.2 53,439,991 rs12185047 Imputed

14q22.2 53,440,346 rs8018146 Genotyped

14q22.2 53,440,499 rs1957848 Genotyped

14q22.2 53,440,935 rs12896755 Imputed

14q22.2 53,441,332 rs10498464 Genotyped

14q22.2 53,441,774 rs59028950 Imputed

14q22.2 53,442,489 rs11845129 Imputed

14q22.2 53,442,591 rs1951865 Genotyped

14q22.2 53,443,042 rs10498465 Imputed

14q22.2 53,444,671 rs12895324 Imputed

14q22.2 53,444,978 rs12879252 Genotyped

14q22.2 53,445,119 rs12879570 Imputed

14q22.2 53,445,177 rs11625471 Imputed

14q22.2 53,445,204 rs11625439 Genotyped

14q22.2 53,453,695 rs11157990 Genotyped

14q22.2 53,453,780 14-53453780 Imputed

14q22.2 53,454,132 rs61985639 Imputed

14q22.2 53,454,407 rs12894409 Imputed

14q22.2 53,455,032 rs72680512 Imputed

14q22.2 53,455,219 rs3742555 Imputed

14q22.2 53,455,740 rs1957850 Imputed

14q22.2 53,456,060 rs12050321 Imputed

14q22.2 53,456,419 rs6572926 Genotyped

14q22.2 53,456,499 rs17127074 Imputed

14q22.2 53,456,565 rs1957851 Imputed

14q22.2 53,456,637 rs12232176 Genotyped

14q22.2 53,457,559 rs1957852 Genotyped

14q22.2 53,457,623 rs4243595 Imputed

14q22.2 53,457,891 rs17832101 Imputed

14q22.2 53,458,043 rs12895146 Imputed

14q22.2 53,458,494 rs12895971 Imputed

14q22.2 53,459,337 rs12323369 Imputed

14q22.2 53,461,392 rs1957856 Imputed

14q22.2 53,461,563 rs10498466 Genotyped

14q22.2 53,461,658 rs7492923 Genotyped

14q22.2 53,462,288 rs35686886 Imputed

14q22.2 53,462,509 rs11157991 Imputed

14q22.2 53,462,594 rs60150385 Imputed

14q22.2 53,463,082 rs10138740 Imputed

14q22.2 53,463,489 rs2147104 Imputed

14q22.2 53,463,762 rs2358429 Imputed

14q22.2 53,463,887 rs4243596 Imputed

14q22.2 53,464,479 rs4901468 Imputed

14q22.2 53,464,481 rs4901469 Imputed

14q22.2 53,464,569 rs4901470 Imputed

14q22.2 53,466,279 rs1957857 Genotyped

14q22.2 53,466,385 rs1957858 Imputed

14q22.2 53,468,503 rs12883570 Genotyped

14q22.2 53,469,007 rs12587398 Imputed

14q22.2 53,469,825 rs942315 Imputed

14q22.2 53,470,173 rs12432196 Imputed

14q22.2 53,471,033 rs8014309 Imputed

14q22.2 53,472,073 14-53472073 Imputed

14q22.2 53,473,871 rs9323252 Imputed

14q22.2 53,475,434 rs9323253 Imputed

14q22.2 53,475,815 rs2147105 Genotyped

14q22.2 53,476,138 rs2181735 Imputed

14q22.2 53,476,491 rs34763890 Imputed

14q22.2 53,476,772 rs2224835 Imputed

14q22.2 53,476,942 rs1951867 Genotyped

14q22.2 53,477,288 14-53477288 Imputed

14q22.2 53,477,396 rs12435446 Imputed

14q22.2 53,477,670 rs7146040 Imputed

14q22.2 53,477,969 rs7151049 Imputed

14q22.2 53,478,333 rs7151613 Imputed

14q22.2 53,478,383 rs1137724 Imputed

14q22.2 53,481,446 rs11157993 Imputed

14q22.2 53,481,836 14-53481836 Imputed

14q22.2 53,482,883 14-53482883 Imputed

14q22.2 53,483,882 rs11623717 Genotyped

14q22.2 53,484,070 14-53484070 Imputed

14q22.2 53,484,488 rs12893484 Imputed

14q22.2 53,485,041 rs12898159 Imputed

14q22.2 53,487,272 rs17563 Genotyped

14q22.2 53,488,161 rs2071047 Genotyped

14q22.2 53,489,302 rs59035991 Imputed

14q22.2 53,492,803 rs762642 Genotyped

14q22.2 53,494,802 rs2761887 Imputed

14q22.2 53,494,897 rs2032423 Imputed

14q22.2 53,495,585 rs1951868 Imputed

14q22.2 53,496,807 rs4898820 Genotyped

14q22.2 53,497,352 rs12434228 Genotyped

14q22.2 53,499,042 rs72680539 Imputed

14q22.2 53,499,105 rs1957860 Genotyped

14q22.2 53,499,535 rs17127103 Imputed

14q22.2 53,500,026 rs72680541 Imputed

14q22.2 53,500,158 14-53500158 Imputed

14q22.2 53,500,595 rs10444719 Genotyped

14q22.2 53,500,877 rs67475977 Imputed

14q22.2 53,501,250 rs8014071 Genotyped

14q22.2 53,501,325 rs8014363 Genotyped

14q22.2 53,502,864 rs7141785 Imputed

14q22.2 53,503,019 rs72680543 Imputed

14q22.2 53,503,140 rs6572927 Genotyped

14q22.2 53,503,283 rs10873077 Genotyped

14q22.2 53,507,352 rs6572929 Imputed

14q22.2 53,509,529 rs9323254 Imputed

14q22.2 53,510,592 rs751399 Imputed

14q22.2 53,510,733 rs942316 Genotyped

14q22.2 53,511,069 rs12895262 Genotyped

14q22.2 53,513,874 rs12892252 Genotyped

14q22.2 53,514,907 rs4901473 Imputed

14q22.2 53,515,693 rs10498467 Imputed

14q22.2 53,517,744 14-53517744 Imputed

14q22.2 53,517,894 14-53517894 Imputed

14q22.2 53,517,896 14-53517896 Imputed

14q22.2 53,517,944 14-53517944 Imputed

14q22.2 53,518,007 rs12590674 Imputed

14q22.2 53,518,034 rs12590643 Imputed

14q22.2 53,518,097 rs12895295 Imputed

14q22.2 53,518,375 rs34931695 Imputed

14q22.2 53,518,621 14-53518621 Imputed

14q22.2 53,518,716 rs1951860 Imputed

14q22.2 53,520,034 rs1957842 Imputed

14q22.2 53,521,052 rs11157994 Imputed

14q22.2 53,521,329 rs1957843 Imputed

14q22.2 53,521,552 rs11157995 Imputed

14q22.2 53,521,644 rs7492912 Imputed

14q22.2 53,521,671 rs61983171 Imputed

14q22.2 53,521,885 rs4281619 Imputed

14q22.2 53,522,171 14-53522171 Imputed

14q22.2 53,522,407 14-53522407 Imputed

14q22.2 53,524,406 14-53524406 Imputed

14q22.2 53,524,987 rs12432350 Imputed

14q22.2 53,525,106 rs55805002 Imputed

14q22.2 53,526,460 rs72680555 Imputed

14q22.2 53,526,981 rs7160450 Genotyped

14q22.2 53,527,210 rs17127124 Genotyped

14q22.2 53,527,828 rs1957844 Genotyped

14q22.2 53,527,877 14-53527877 Imputed

14q22.2 53,527,909 rs1957845 Genotyped

14q22.2 53,528,033 14-53528033 Imputed

14q22.2 53,528,742 14-53528742 Imputed

14q22.2 53,528,760 rs7149231 Imputed

14q22.2 53,528,784 rs7150572 Imputed

14q22.2 53,528,954 rs7149575 Imputed

14q22.2 53,530,761 rs17127134 Genotyped

**Region Location SNP ID Imputed or Genotyped**

20p12.3 6,292,730 rs6085517 Genotyped

20p12.3 6,293,198 20-6293198 Imputed

20p12.3 6,293,709 rs6085518 Genotyped

20p12.3 6,294,430 20-6294430 Imputed

20p12.3 6,294,620 rs58646818 Imputed

20p12.3 6,296,176 rs62200799 Imputed

20p12.3 6,296,251 rs6054166 Imputed

20p12.3 6,298,827 rs61256347 Imputed

20p12.3 6,299,267 rs6054177 Imputed

20p12.3 6,299,417 rs6076987 Genotyped

20p12.3 6,300,470 rs2148813 Imputed

20p12.3 6,300,589 rs2148814 Imputed

20p12.3 6,302,161 rs6054183 Imputed

20p12.3 6,302,321 rs62200805 Imputed

20p12.3 6,302,592 rs6054184 Imputed

20p12.3 6,302,914 rs2182709 Imputed

20p12.3 6,303,181 rs6139973 Imputed

20p12.3 6,303,395 rs6133310 Imputed

20p12.3 6,304,246 rs6085521 Imputed

20p12.3 6,305,707 rs6085522 Genotyped

20p12.3 6,305,758 rs6085523 Imputed

20p12.3 6,307,041 rs17792746 Imputed

20p12.3 6,307,474 rs17719553 Genotyped

20p12.3 6,308,394 rs6038468 Imputed

20p12.3 6,308,465 rs2225115 Genotyped

20p12.3 6,308,748 rs6139974 Imputed

20p12.3 6,309,009 rs73082401 Imputed

20p12.3 6,309,273 rs6085525 Genotyped

20p12.3 6,309,948 rs13039230 Genotyped

20p12.3 6,310,730 rs959278 Imputed

20p12.3 6,311,207 rs2182710 Genotyped

20p12.3 6,311,480 rs2148815 Imputed

20p12.3 6,312,504 rs2209760 Genotyped

20p12.3 6,312,637 rs2025837 Imputed

20p12.3 6,313,190 rs2182711 Genotyped

20p12.3 6,313,645 rs6054200 Imputed

20p12.3 6,314,902 rs6076988 Imputed

20p12.3 6,314,990 rs6085527 Imputed

20p12.3 6,315,087 rs6139975 Imputed

20p12.3 6,316,089 rs2182712 Genotyped

20p12.3 6,318,053 rs1571218 Genotyped

20p12.3 6,318,283 rs6076990 Imputed

20p12.3 6,319,011 rs2209761 Genotyped

20p12.3 6,319,246 rs2209762 Imputed

20p12.3 6,319,396 rs6076991 Imputed

20p12.3 6,319,490 rs6085530 Imputed

20p12.3 6,319,505 rs6054204 Imputed

20p12.3 6,319,974 rs1411300 Imputed

20p12.3 6,321,187 rs355533 Imputed

20p12.3 6,321,935 rs355531 Imputed

20p12.3 6,322,170 rs355530 Imputed

20p12.3 6,322,388 rs355529 Imputed

20p12.3 6,323,097 rs2876019 Imputed

20p12.3 6,323,158 rs2876020 Imputed

20p12.3 6,323,460 rs6085533 Imputed

20p12.3 6,324,017 rs355528 Genotyped

20p12.3 6,324,457 rs189583 Genotyped

20p12.3 6,325,085 rs419165 Imputed

20p12.3 6,325,183 rs6085534 Imputed

20p12.3 6,325,293 rs450007 Imputed

20p12.3 6,325,334 rs384409 Imputed

20p12.3 6,325,396 rs426027 Imputed

20p12.3 6,325,542 rs431755 Imputed

20p12.3 6,325,629 rs12625146 Imputed

20p12.3 6,325,904 rs432106 Imputed

20p12.3 6,325,965 rs432266 Imputed

20p12.3 6,325,979 rs432277 Imputed

20p12.3 6,326,180 rs441147 Imputed

20p12.3 6,326,672 rs438030 Imputed

20p12.3 6,326,673 rs437708 Imputed

20p12.3 6,326,809 rs445074 Imputed

20p12.3 6,326,890 rs438009 Imputed

20p12.3 6,326,912 rs445218 Imputed

20p12.3 6,327,635 rs453114 Imputed

20p12.3 6,328,001 rs654408 Imputed

20p12.3 6,328,022 rs654433 Imputed

20p12.3 6,328,036 rs542134 Imputed

20p12.3 6,328,246 rs433414 Imputed

20p12.3 6,328,323 rs446391 Imputed

20p12.3 6,328,344 rs57046232 Imputed

20p12.3 6,328,461 rs7275115 Imputed

20p12.3 6,328,637 rs373038 Imputed

20p12.3 6,329,216 rs2423149 Imputed

20p12.3 6,329,666 rs6038478 Imputed

20p12.3 6,329,747 rs2209763 Imputed

20p12.3 6,329,804 rs6085539 Imputed

20p12.3 6,329,955 rs11087729 Imputed

20p12.3 6,330,002 rs36026248 Imputed

20p12.3 6,330,301 rs913245 Imputed

20p12.3 6,330,377 rs6085541 Imputed

20p12.3 6,330,668 rs6085542 Imputed

20p12.3 6,331,303 rs6038479 Imputed

20p12.3 6,331,469 rs62198487 Imputed

20p12.3 6,331,527 rs2423151 Genotyped

20p12.3 6,331,710 rs17720145 Genotyped

20p12.3 6,334,243 rs6076994 Imputed

20p12.3 6,334,438 rs35469553 Imputed

20p12.3 6,336,068 rs355527 Genotyped

20p12.3 6,336,310 rs62198489 Imputed

20p12.3 6,336,401 rs355526 Imputed

20p12.3 6,337,167 rs6076995 Imputed

20p12.3 6,338,976 rs28665621 Imputed

20p12.3 6,340,251 20-6340251 Imputed

20p12.3 6,340,520 rs2209751 Imputed

20p12.3 6,340,681 rs2423154 Imputed

20p12.3 6,341,733 rs73084196 Imputed

20p12.3 6,342,405 rs62198490 Imputed

20p12.3 6,342,652 rs2423156 Imputed

20p12.3 6,343,353 rs438945 Imputed

20p12.3 6,344,379 rs355525 Imputed

20p12.3 6,344,978 rs6054220 Imputed

20p12.3 6,344,990 rs2423157 Imputed

20p12.3 6,345,036 rs6038483 Genotyped

20p12.3 6,345,236 rs2423158 Imputed

20p12.3 6,345,292 rs2423159 Imputed

20p12.3 6,345,323 rs173322 Imputed

20p12.3 6,346,058 20-6346058 Imputed

20p12.3 6,346,127 rs6085545 Imputed

20p12.3 6,346,192 rs6133312 Genotyped

20p12.3 6,346,572 rs1115460 Imputed

20p12.3 6,346,949 rs11905859 Genotyped

20p12.3 6,347,494 rs2876032 Imputed

20p12.3 6,347,875 rs355524 Genotyped

20p12.3 6,347,890 rs1998970 Imputed

20p12.3 6,348,688 rs62198491 Imputed

20p12.3 6,349,824 rs355523 Imputed

20p12.3 6,350,213 rs16992177 Genotyped

20p12.3 6,350,483 rs6117250 Imputed

20p12.3 6,350,559 rs2423160 Imputed

20p12.3 6,350,641 rs355522 Imputed

20p12.3 6,351,132 rs355521 Imputed

20p12.3 6,351,218 rs73086114 Imputed

20p12.3 6,351,232 20-6351232 Imputed

20p12.3 6,351,267 rs355520 Imputed

20p12.3 6,351,431 rs17793475 Imputed

20p12.3 6,351,521 rs8124724 Imputed

20p12.3 6,351,639 rs62198492 Imputed

20p12.3 6,351,774 rs6516161 Imputed

20p12.3 6,351,804 rs6516162 Genotyped

20p12.3 6,351,919 rs6516163 Imputed

20p12.3 6,351,930 rs6516164 Imputed

20p12.3 6,352,117 rs4292146 Imputed

20p12.3 6,352,364 rs961254 Imputed

20p12.3 6,352,442 rs5005940 Imputed

20p12.3 6,353,044 rs4815894 Imputed

20p12.3 6,353,408 rs13045451 Imputed

20p12.3 6,353,479 rs13037538 Imputed

20p12.3 6,353,607 rs6054223 Imputed

20p12.3 6,353,614 rs62198536 Imputed

20p12.3 6,353,744 rs71338563 Imputed

20p12.3 6,353,944 rs71338564 Imputed

20p12.3 6,354,102 rs6085549 Genotyped

20p12.3 6,354,440 rs6117251 Genotyped

20p12.3 6,354,554 rs7264777 Genotyped

20p12.3 6,355,326 rs6133313 Genotyped

20p12.3 6,355,642 rs6139980 Genotyped

20p12.3 6,355,912 20-6355912 Imputed

20p12.3 6,355,950 rs6076999 Imputed

20p12.3 6,356,094 rs6085550 Genotyped

20p12.3 6,356,095 rs61010364 Imputed

20p12.3 6,356,114 rs6077000 Imputed

20p12.3 6,356,618 rs6417634 Imputed

20p12.3 6,356,948 rs6054226 Imputed

20p12.3 6,357,183 20-6357183 Imputed

20p12.3 6,358,055 rs4815896 Imputed

20p12.3 6,358,235 rs6117253 Imputed

20p12.3 6,358,370 rs62198542 Imputed

20p12.3 6,358,854 rs6038491 Genotyped

20p12.3 6,359,069 rs4815897 Genotyped

20p12.3 6,359,203 rs6139981 Genotyped

20p12.3 6,359,560 rs6139982 Genotyped

20p12.3 6,361,582 rs955403 Imputed

20p12.3 6,361,830 rs2326783 Imputed

20p12.3 6,361,941 rs2225345 Imputed

20p12.3 6,362,773 rs6117256 Genotyped

20p12.3 6,363,093 rs11905377 Imputed

20p12.3 6,363,539 rs6107793 Imputed

20p12.3 6,363,715 rs6054230 Imputed

20p12.3 6,363,824 rs6054232 Imputed

20p12.3 6,364,132 rs953420 Imputed

20p12.3 6,364,462 rs6107795 Imputed

20p12.3 6,364,464 rs2225346 Imputed

20p12.3 6,364,544 rs6107796 Imputed

20p12.3 6,364,883 rs7265115 Genotyped

20p12.3 6,364,991 rs6139983 Imputed

20p12.3 6,364,994 rs6139984 Imputed

20p12.3 6,365,028 rs6139985 Imputed

20p12.3 6,365,299 rs2094802 Imputed

20p12.3 6,365,695 rs2149951 Imputed

20p12.3 6,365,802 rs2149950 Imputed

20p12.3 6,366,188 rs4280520 Imputed

20p12.3 6,366,645 rs6139990 Imputed

20p12.3 6,366,776 rs6139991 Imputed

20p12.3 6,366,814 rs6139992 Imputed

20p12.3 6,366,899 rs2026168 Imputed

20p12.3 6,367,194 rs6077001 Imputed

20p12.3 6,367,300 rs1888254 Imputed

20p12.3 6,367,320 rs1888255 Imputed

20p12.3 6,367,378 rs6117258 Imputed

20p12.3 6,367,793 rs62198543 Imputed

20p12.3 6,368,731 rs6117259 Genotyped

20p12.3 6,369,173 rs6054239 Imputed

20p12.3 6,369,211 rs6054240 Imputed

20p12.3 6,369,903 rs62199969 Imputed

20p12.3 6,370,001 rs6038495 Genotyped

20p12.3 6,370,103 rs6038497 Imputed

20p12.3 6,370,123 rs13042469 Imputed

20p12.3 6,370,519 rs6038498 Imputed

20p12.3 6,370,570 rs6139993 Imputed

20p12.3 6,370,606 rs6139994 Imputed

20p12.3 6,370,658 rs6054242 Imputed

20p12.3 6,370,697 rs6117260 Genotyped

20p12.3 6,370,948 rs996773 Imputed

20p12.3 6,371,044 rs62199971 Imputed

20p12.3 6,371,485 rs4083664 Imputed

20p12.3 6,371,578 rs6139995 Genotyped

20p12.3 6,371,857 rs62199972 Imputed

20p12.3 6,372,296 rs6054243 Imputed

20p12.3 6,372,684 rs6054245 Imputed

20p12.3 6,372,857 rs6054248 Imputed

20p12.3 6,373,158 rs6117261 Imputed

20p12.3 6,373,899 rs6085559 Imputed

20p12.3 6,373,967 rs6117262 Imputed

20p12.3 6,374,137 rs6133317 Genotyped

20p12.3 6,375,039 rs2225347 Imputed

20p12.3 6,375,132 rs6054251 Imputed

20p12.3 6,375,177 rs6054252 Imputed

20p12.3 6,375,241 rs6054253 Imputed

20p12.3 6,375,518 rs6054255 Genotyped

20p12.3 6,376,354 rs6038501 Imputed

20p12.3 6,376,622 rs1156511 Imputed

20p12.3 6,376,664 rs1156510 Imputed

20p12.3 6,376,704 rs1156509 Imputed

20p12.3 6,376,764 rs4815900 Genotyped

20p12.3 6,377,864 rs6085561 Imputed

20p12.3 6,377,964 rs6117263 Imputed

20p12.3 6,378,018 rs6054258 Imputed

20p12.3 6,378,089 rs6085562 Imputed

20p12.3 6,378,101 rs11087731 Genotyped

20p12.3 6,378,159 rs6054259 Imputed

20p12.3 6,378,197 rs6054260 Imputed

20p12.3 6,378,551 rs6054261 Imputed

20p12.3 6,378,556 rs13038613 Imputed

20p12.3 6,378,639 rs6139998 Imputed

20p12.3 6,378,751 rs6054262 Imputed

20p12.3 6,378,769 rs6054263 Imputed

20p12.3 6,378,884 rs6107805 Imputed

20p12.3 6,379,104 rs6133319 Imputed

20p12.3 6,379,267 rs6054264 Imputed

20p12.3 6,379,316 rs953959 Genotyped

20p12.3 6,379,393 rs6054265 Imputed

20p12.3 6,379,457 rs6054266 Imputed

20p12.3 6,379,569 rs6054267 Genotyped

20p12.3 6,379,625 rs2326784 Imputed

20p12.3 6,379,823 rs2210122 Imputed

20p12.3 6,379,914 rs6054268 Imputed

20p12.3 6,380,075 rs6054269 Imputed

20p12.3 6,380,432 rs6054271 Imputed

20p12.3 6,380,447 rs10775627 Imputed

20p12.3 6,380,618 rs6054272 Imputed

20p12.3 6,380,735 rs2876033 Imputed

20p12.3 6,380,797 rs2326785 Imputed

20p12.3 6,381,261 rs6054274 Imputed

20p12.3 6,381,489 rs6038504 Imputed

20p12.3 6,381,765 rs6038505 Imputed

20p12.3 6,381,854 rs6038506 Imputed

20p12.3 6,382,050 rs6038507 Imputed

20p12.3 6,382,117 rs6054275 Imputed

20p12.3 6,382,266 rs2225348 Genotyped

20p12.3 6,382,843 rs6038509 Imputed

20p12.3 6,383,029 rs6038510 Imputed

20p12.3 6,383,557 rs6054276 Imputed

20p12.3 6,383,558 rs6054277 Imputed

20p12.3 6,383,616 rs6117264 Imputed

20p12.3 6,383,676 rs6054278 Imputed

20p12.3 6,383,698 rs6038511 Imputed

20p12.3 6,383,839 rs6140000 Genotyped

20p12.3 6,383,967 rs2210124 Imputed

20p12.3 6,384,817 rs6117265 Imputed

20p12.3 6,384,838 rs6107806 Imputed

20p12.3 6,385,727 rs6038513 Imputed

20p12.3 6,385,981 rs6054282 Imputed

20p12.3 6,386,000 20-6386000 Imputed

20p12.3 6,386,282 rs13042028 Imputed

20p12.3 6,386,553 rs2183450 Imputed

20p12.3 6,386,797 rs6038514 Imputed

20p12.3 6,387,088 rs6054284 Imputed

20p12.3 6,388,258 rs7509140 Imputed

20p12.3 6,390,961 rs6077004 Genotyped

20p12.3 6,392,659 rs13036856 Imputed

20p12.3 6,392,931 rs7269050 Genotyped

20p12.3 6,393,632 rs62199997 Imputed

20p12.3 6,393,985 rs6054289 Genotyped

20p12.3 6,394,135 rs58021936 Imputed

20p12.3 6,394,150 rs6117271 Imputed

20p12.3 6,394,162 rs6117272 Genotyped

20p12.3 6,394,350 rs16992249 Imputed

20p12.3 6,395,536 rs6117273 Imputed

20p12.3 6,395,663 rs12626134 Imputed

20p12.3 6,396,094 rs7262110 Imputed

20p12.3 6,396,432 rs6117275 Imputed

20p12.3 6,396,678 rs6117277 Genotyped

20p12.3 6,396,768 rs6117278 Imputed

20p12.3 6,397,228 rs6085565 Imputed

20p12.3 6,397,281 rs6085566 Imputed

20p12.3 6,397,722 rs62199999 Imputed

20p12.3 6,398,549 rs990123 Imputed

20p12.3 6,399,752 rs2326787 Genotyped

20p12.3 6,400,601 rs6054290 Imputed

20p12.3 6,400,773 rs6054291 Genotyped

20p12.3 6,400,982 rs6085568 Genotyped

20p12.3 6,401,105 rs6085569 Imputed

20p12.3 6,401,324 rs4815903 Genotyped

20p12.3 6,402,309 rs6077007 Genotyped

20p12.3 6,402,661 rs6054292 Genotyped
