# Supplementary material for: Multiple Common Susceptibility Variants near BMP Pathway Loci GREM1, BMP4, and BMP2 Explain Part of the Missing Heritability of Colorectal Cancer
Source: PLoS Genet. 2011 Jun 2;7(6):e1002105. doi: 10.1371/journal.pgen.1002105 (PMC3107194; doi:10.1371/journal.pgen.1002105)
Supplement: Table S2 — Haplotype risk analysis at rs16969681 and rs4779584. (DOCX) [file pgen.1002105.s008.docx]

*Supplemental Table 2. Haplotype risk analysis at rs16969681 and rs4779584.*

Haploview (http://[www.haploview.org](http://www.haploview.org)/) was used to estimate haplotype frequencies in our data at SNPs close to rs16969681 and rs4779584. We then used PLINK to perform haplotype association analysis at rs16969681 and rs4779584.

LOCUS HAPLOTYPE F_A F_U CHISQ DF P SNPs

WIN1 OMNIBUS NA NA 24.91 3 1.609x10^-5^ rs16969681|rs4779584

WIN1 TT 0.0632 0.0525 16.01 1 6.29x10^-5^ rs16969681|rs4779584

WIN1 CT 0.1470 0.1380 4.938 1 0.02628 rs16969681|rs4779584

WIN1 TC 0.0326 0.0306 1.008 1 0.3154 rs16969681|rs4779584

WIN1 CC 0.7571 0.7789 19.94 1 7.974x10^-6^ rs16969681|rs4779584
