# Supplementary material for: Multiple Common Susceptibility Variants near BMP Pathway Loci GREM1, BMP4, and BMP2 Explain Part of the Missing Heritability of Colorectal Cancer
Source: PLoS Genet. 2011 Jun 2;7(6):e1002105. doi: 10.1371/journal.pgen.1002105 (PMC3107194; doi:10.1371/journal.pgen.1002105)
Supplement: Table S3 — Logistic regression model analysis of CRC risk and genotypes at rs4779584, rs16969681, and rs11632715. (DOCX) [file pgen.1002105.s009.docx]

*Supplemental Table 3. Logistic regression model analysis of CRC risk and genotypes at rs4779584, rs16969681 and rs11632715.*

Samples typed for all 3 SNPs are included. The best fitting model (minimum Akaike Information Criterion) included rs16969681 and rs11632715. Inclusion of rs4779584 did not improve the fit of the model. When all 3 SNPs were included in the logistic regression model: for rs11632715, P=1.36x10^-5^; for rs16969681, P=3.96x10^-5^; and for rs4779584, P=0.14.

SNPs included in model AIC__

rs4779584+rs16969681+rs11632715 25541

rs4779584+rs16969681 25558

rs4779584+rs11632715 25556

rs16969681+rs11632715 25541

rs4779584 25570

rs16969681 25565

rs11632715 25564

Although the data for the SNPs near *GREM1* are by far most compatible with 2 independent CRC variants at or tagged by rs16969681 and rs11632715, we cannot entirely exclude the remote possibility of the signals at rs16969681 and rs11632715 capturing a single very rare disease variant on a haplotype which we estimate to have a frequency of ~1%. However, no such variant was evident on imputation using 1000 genomes project data as a reference (see above). Moreover, such a variant might have a sufficiently large effect size that it would be detectable by linkage screens, and no evidence of a such a linkage signal at *GREM1* has emerged from linkage studies of CRC in white northern European populations (2-7). It is also highly implausible that these SNPs are reporting the effects of the high-penetrance *HMPS/CRAC1* gene found in Ashkenazim (8), since HMPS families are generally large, have distinct phenotypes and tumour histology. Moreover, the *HMPS/CRAC1* ancestral mutation resides on a haplotype that does not include the risk allele at rs16969681 or rs11632715.
