# Supplementary material for: Multiple Common Susceptibility Variants near BMP Pathway Loci GREM1, BMP4, and BMP2 Explain Part of the Missing Heritability of Colorectal Cancer
Source: PLoS Genet. 2011 Jun 2;7(6):e1002105. doi: 10.1371/journal.pgen.1002105 (PMC3107194; doi:10.1371/journal.pgen.1002105)
Supplement: Table S4 — TagSNPs around GREM1, BMP4, and BMP2 analysed for new associations. (DOCX) [file pgen.1002105.s010.docx]

*Supplemental Table 4. TagSNPs around GREM1, BMP4 and BMP2 analysed for new associations.*

**CHR BP SNP**

15q13.3 30,775,388 rs1406388

15q13.3 30,775,430 rs7494781

15q13.3 30,779,005 rs16969344

15q13.3 30,779,779 rs12708490

15q13.3 30,780,036 rs8035130

15q13.3 30,780,403 rs16969681

15q13.3 30,780,937 rs16969816

15q13.3 30,781,293 rs16969862

15q13.3 30,781,348 rs12591992

15q13.3 30,781,704 rs12592056

15q13.3 30,781,917 rs12594722

15q13.3 30,782,048 rs4779584

15q13.3 30,782,135 rs9888701

15q13.3 30,782,198 rs7172208

15q13.3 30,782,590 rs16970016

15q13.3 30,782,673 rs7166282

15q13.3 30,784,442 rs3861195

15q13.3 30,784,874 rs11853552

15q13.3 30,785,128 rs11857997

15q13.3 30,787,098 rs1554865

15q13.3 30,788,001 rs1881538

15q13.3 30,788,556 rs12914734

15q13.3 30,788,858 rs7182252

15q13.3 30,791,354 rs11638007

15q13.3 30,791,539 rs11632715

15q13.3 30,793,167 rs1534594

15q13.3 30,793,800 rs12592288

15q13.3 30,796,770 rs1406389

15q13.3 30,798,028 rs2293581

15q13.3 30,802,498 rs1528734

15q13.3 30,802,694 rs7497354

15q13.3 30,802,752 rs9920024

15q13.3 30,803,446 rs11630554

15q13.3 30,803,770 rs10519738

15q13.3 30,808,220 rs16973303

15q13.3 30,809,874 rs11854391

15q13.3 30,810,390 rs2280738

15q13.3 30,810,778 rs12915554

15q13.3 30,811,243 rs3743105

15q13.3 30,811,747 rs7162202

15q13.3 30,811,859 rs17525764

15q13.3 30,811,859 rs17816279

15q13.3 30,812,919 rs3743103

15q13.3 30,813,271 rs10318

15q13.3 30,813,959 rs1129456

15q13.3 30,814,471 rs7176378

15q13.3 30,816,842 rs11071936

15q13.3 30,820,130 rs4453446

15q13.3 30,820,399 rs7175986

15q13.3 30,824,409 rs12905295

15q13.3 30,825,787 rs7496435

15q13.3 30,826,590 rs17816285

15q13.3 30,827,888 rs10519739

15q13.3 30,828,074 rs10519740

15q13.3 30,828,286 rs12439770

15q13.3 30,829,630 rs16958114

15q13.3 30,830,125 rs12904470

15q13.3 30,830,747 rs1919360

15q13.3 30,830,949 rs1919362

15q13.3 30,835,148 rs11633236

15q13.3 30,837,715 rs1258763

15q13.3 30,839,102 rs1258756

15q13.3 30,840,760 rs12050872

15q13.3 30,841,293 rs2697937

15q13.3 30,842,858 rs3108628

15q13.3 30,843,196 rs2141438

15q13.3 30,843,508 rs2053939

15q13.3 30,843,508 rs4482251

15q13.3 30,844,649 rs1258721

15q13.3 30,845,067 rs3817591

15q13.3 30,845,815 rs12148620

15q13.3 30,846,355 rs3812929

15q13.3 30,847,699 rs2840190

15q13.3 30,847,893 rs1020561

15q13.3 30,848,487 rs1258724

15q13.3 30,848,692 rs11853746

15q13.3 30,849,208 rs1258726

15q13.3 30,849,631 rs3110558

15q13.3 30,849,653 rs12442768

15q13.3 30,849,684 rs16958617

15q13.3 30,850,170 rs1979167

15q13.3 30,850,518 rs1979168

15q13.3 30,851,101 rs3743106

15q13.3 30,851,408 rs3743108

15q13.3 30,851,440 rs3825862

15q13.3 30,851,589 rs17816333

15q13.3 30,852,083 rs1258731

15q13.3 30,852,603 rs1258732

15q13.3 30,852,937 rs2037844

15q13.3 30,853,102 rs16958702

15q13.3 30,854,631 rs17816345

15q13.3 30,854,948 rs1258734

15q13.3 30,855,275 rs1258735

15q13.3 30,855,315 rs1258736

15q13.3 30,855,341 rs12594522

15q13.3 30,855,573 rs896507

15q13.3 30,861,143 rs2077680

**CHR BP SNP**

14q22.2 52,555,108 rs4901334

14q22.2 52,558,141 rs12433730

14q22.2 52,563,324 rs10137117

14q22.2 52,563,872 rs10139750

14q22.2 52,563,901 rs8021587

14q22.2 52,569,647 rs17126068

14q22.2 52,572,713 rs12897244

14q22.2 52,575,641 rs2150541

14q22.2 52,577,434 rs17126072

14q22.2 52,595,177 rs12882412

14q22.2 52,612,077 rs2152492

14q22.2 52,695,157 rs8005652

14q22.2 52,701,283 rs10483621

14q22.2 52,701,921 rs4901357

14q22.2 52,711,951 rs7160009

14q22.2 52,730,313 rs2552400

14q22.2 52,735,601 rs2254182

14q22.2 52,752,572 rs4901365

14q22.2 52,758,455 rs6572885

14q22.2 52,763,499 rs1270515

14q22.2 52,770,091 rs11157957

14q22.2 52,772,120 rs1424832

14q22.2 52,776,759 rs763328

14q22.2 52,801,683 rs7146135

14q22.2 52,810,756 rs7151631

14q22.2 52,814,615 rs1364597

14q22.2 52,823,675 rs8013473

14q22.2 52,830,063 rs2161944

14q22.2 52,830,264 rs12434333

14q22.2 52,831,455 rs11626176

14q22.2 52,833,496 rs4901392

14q22.2 52,840,394 rs10132541

14q22.2 52,862,629 rs4901403

14q22.2 52,867,043 rs17126346

14q22.2 52,867,898 rs1424838

14q22.2 52,871,996 rs17126349

14q22.2 52,873,879 rs4901408

14q22.2 52,874,082 rs8023182

14q22.2 52,881,770 rs17126361

14q22.2 52,883,170 rs1255329

14q22.2 52,886,214 rs17126390

14q22.2 52,889,307 rs1255311

14q22.2 52,889,727 rs1255309

14q22.2 52,892,776 rs877018

14q22.2 52,896,997 rs1255288

14q22.2 52,903,676 rs1255277

14q22.2 52,908,879 rs17126432

14q22.2 52,912,225 rs17831927

14q22.2 52,913,714 rs6572905

14q22.2 52,918,686 rs4901416

14q22.2 52,919,582 rs1959839

14q22.2 52,929,105 rs3951266

14q22.2 52,931,563 rs2185823

14q22.2 52,931,636 rs7145276

14q22.2 52,936,440 rs7152432

14q22.2 52,938,344 rs1954329

14q22.2 52,938,563 rs12881359

14q22.2 52,941,665 rs1959844

14q22.2 52,942,144 rs12147371

14q22.2 52,942,157 rs10137772

14q22.2 52,943,242 rs7140955

14q22.2 52,943,591 rs10141685

14q22.2 52,945,697 rs8016132

14q22.2 52,945,741 rs8020203

14q22.2 52,946,599 rs12896513

14q22.2 52,947,864 rs11622595

14q22.2 52,949,420 rs12588025

14q22.2 52,949,813 rs1959846

14q22.2 52,956,528 rs11157969

14q22.2 52,958,888 rs1954332

14q22.2 52,976,589 rs11846138

14q22.2 52,977,625 rs2358222

14q22.2 52,978,083 rs1891552

14q22.2 52,981,315 rs17253256

14q22.2 52,982,078 rs11623604

14q22.2 52,982,584 rs1954333

14q22.2 52,989,059 rs1954303

14q22.2 52,989,928 rs11849931

14q22.2 52,992,995 rs7146329

14q22.2 53,008,870 rs4901426

14q22.2 53,012,538 rs17126556

14q22.2 53,012,683 rs17126559

14q22.2 53,020,233 rs1954313

14q22.2 53,021,754 rs12437319

14q22.2 53,028,619 rs2358227

14q22.2 53,029,970 rs12433690

14q22.2 53,049,564 rs4901431

14q22.2 53,050,472 rs17093060

14q22.2 53,053,482 rs12896283

14q22.2 53,054,715 rs11157973

14q22.2 53,056,970 rs2000224

14q22.2 53,061,954 rs2251492

14q22.2 53,070,047 rs1891550

14q22.2 53,076,776 rs2245007

14q22.2 53,082,853 rs11157975

14q22.2 53,090,485 rs10145210

14q22.2 53,097,749 rs4584745

14q22.2 53,097,749 rs7493781

14q22.2 53,111,705 rs1999345

14q22.2 53,116,516 rs17705765

14q22.2 53,117,621 rs2810061

14q22.2 53,118,724 rs1984025

14q22.2 53,120,826 rs7152946

14q22.2 53,125,087 rs7156227

14q22.2 53,126,377 rs1001161

14q22.2 53,126,732 rs2776514

14q22.2 53,138,531 rs210327

14q22.2 53,140,662 rs210326

14q22.2 53,142,608 rs1380131

14q22.2 53,159,106 rs10873073

14q22.2 53,159,356 rs10483624

14q22.2 53,159,827 rs10483623

14q22.2 53,161,879 rs4901434

14q22.2 53,165,583 rs210375

14q22.2 53,169,450 rs210388

14q22.2 53,171,619 rs8020341

14q22.2 53,171,759 rs210386

14q22.2 53,172,763 rs6572916

14q22.2 53,177,541 rs210381

14q22.2 53,188,522 rs210321

14q22.2 53,193,102 rs210313

14q22.2 53,193,501 rs210311

14q22.2 53,202,404 rs10141869

14q22.2 53,212,776 rs7154592

14q22.2 53,214,603 rs7140911

14q22.2 53,215,067 rs7146962

14q22.2 53,218,192 rs17126761

14q22.2 53,223,746 rs210302

14q22.2 53,227,716 rs210370

14q22.2 53,231,830 rs210363

14q22.2 53,233,786 rs210361

14q22.2 53,234,456 rs210360

14q22.2 53,237,222 rs210359

14q22.2 53,237,867 rs964254

14q22.2 53,239,490 rs210357

14q22.2 53,242,605 rs7151053

14q22.2 53,243,289 rs210352

14q22.2 53,245,589 rs12885035

14q22.2 53,247,211 rs1026487

14q22.2 53,248,640 rs17635121

14q22.2 53,248,686 rs8005983

14q22.2 53,250,349 rs210343

14q22.2 53,255,833 rs169748

14q22.2 53,256,814 rs7155858

14q22.2 53,256,878 rs17126825

14q22.2 53,262,222 rs210332

14q22.2 53,272,873 rs10143898

14q22.2 53,274,830 rs1380124

14q22.2 53,275,362 rs12895682

14q22.2 53,275,921 rs12586452

14q22.2 53,276,387 rs11621375

14q22.2 53,280,536 rs9323246

14q22.2 53,281,917 rs8009949

14q22.2 53,286,519 rs11623166

14q22.2 53,292,038 rs12590186

14q22.2 53,294,303 rs6572922

14q22.2 53,295,858 rs4901444

14q22.2 53,296,123 rs12894060

14q22.2 53,296,438 rs12184995

14q22.2 53,298,267 rs1958654

14q22.2 53,298,796 rs8015573

14q22.2 53,304,503 rs10147611

14q22.2 53,308,392 rs10498453

14q22.2 53,308,747 rs10498454

14q22.2 53,313,634 rs4901450

14q22.2 53,313,634 rs7401585

14q22.2 53,316,103 rs12589674

14q22.2 53,317,833 rs17126895

14q22.2 53,321,945 rs7154006

14q22.2 53,322,290 rs883519

14q22.2 53,327,510 rs17126903

14q22.2 53,329,348 rs8017615

14q22.2 53,333,601 rs8009420

14q22.2 53,340,961 rs8004644

14q22.2 53,343,338 rs2150279

14q22.2 53,344,193 rs8020936

14q22.2 53,347,739 rs17126953

14q22.2 53,352,059 rs11849392

14q22.2 53,360,138 rs2884439

14q22.2 53,360,547 rs12434693

14q22.2 53,360,580 rs12431733

14q22.2 53,361,132 rs4898812

14q22.2 53,362,407 rs883219

14q22.2 53,365,399 rs1958636

14q22.2 53,371,277 rs7154026

14q22.2 53,372,596 rs4901454

14q22.2 53,380,347 rs11157980

14q22.2 53,380,675 rs1952748

14q22.2 53,380,777 rs2150276

14q22.2 53,386,357 rs7148896

14q22.2 53,390,192 rs1958643

14q22.2 53,391,034 rs1958644

14q22.2 53,391,156 rs1958645

14q22.2 53,398,432 rs1888344

14q22.2 53,400,045 rs4901458

14q22.2 53,409,243 rs7160860

14q22.2 53,410,376 rs7156353

14q22.2 53,415,547 rs17127017

14q22.2 53,427,887 rs17127035

14q22.2 53,437,350 rs12435627

14q22.2 53,438,622 rs2181733

14q22.2 53,440,346 rs8018146

14q22.2 53,441,332 rs10498464

14q22.2 53,442,591 rs1951865

14q22.2 53,444,978 rs12879252

14q22.2 53,445,204 rs11625439

14q22.2 53,453,695 rs11157990

14q22.2 53,461,563 rs10498466

14q22.2 53,475,815 rs2147105

14q22.2 53,480,669 rs4444235

14q22.2 53,487,272 rs17563

14q22.2 53,488,161 rs2071047

14q22.2 53,492,803 rs762642

14q22.2 53,497,352 rs12434228

14q22.2 53,499,105 rs1957860

14q22.2 53,501,325 rs8014363

14q22.2 53,503,283 rs10873077

14q22.2 53,507,352 rs6572929

14q22.2 53,513,874 rs12892252

14q22.2 53,521,052 rs11157994

14q22.2 53,526,981 rs7160450

14q22.2 53,527,828 rs1957844

14q22.2 53,531,911 rs17253452

14q22.2 53,533,084 rs7141833

14q22.2 53,539,487 rs4901474

14q22.2 53,543,549 rs7149949

14q22.2 53,576,486 rs17708795

14q22.2 53,583,027 rs1983786

14q22.2 53,588,576 rs11158001

14q22.2 53,589,723 rs1951659

14q22.2 53,592,607 rs1033823

14q22.2 53,597,817 rs11621185

14q22.2 53,616,199 rs8007000

14q22.2 53,619,256 rs12878931

14q22.2 53,629,768 rs1957636

14q22.2 53,643,881 rs12587564

14q22.2 53,651,669 rs713423

14q22.2 53,660,900 rs10483627

14q22.2 53,664,028 rs996041

14q22.2 53,669,837 rs4898828

14q22.2 53,674,026 rs17127321

14q22.2 53,677,386 rs2358477

14q22.2 53,677,512 rs811165

14q22.2 53,689,066 rs811153

14q22.2 53,699,691 rs932683

14q22.2 53,710,314 rs8012016

14q22.2 53,716,500 rs10131289

14q22.2 53,722,229 rs1953743

14q22.2 53,728,957 rs8008441

14q22.2 53,738,263 rs4901494

14q22.2 53,753,737 rs10134051

14q22.2 53,763,218 rs1187428

14q22.2 53,766,489 rs10133577

14q22.2 53,766,574 rs11627070

14q22.2 53,766,956 rs1187430

14q22.2 53,769,416 rs1187432

14q22.2 53,769,745 rs11629285

14q22.2 53,793,804 rs2358483

14q22.2 53,800,017 rs4901501

14q22.2 53,804,500 rs1008499

14q22.2 53,822,058 rs10143156

14q22.2 53,825,276 rs2179897

14q22.2 53,826,585 rs4287470

14q22.2 53,827,247 rs7359097

14q22.2 53,830,397 rs6572944

14q22.2 53,831,209 rs1950208

14q22.2 53,835,243 rs11845885

14q22.2 53,838,647 rs12432299

14q22.2 53,838,761 rs12432843

14q22.2 53,839,589 rs6572946

14q22.2 53,842,379 rs1884015

14q22.2 53,843,761 rs1950210

14q22.2 53,844,684 rs9323260

14q22.2 53,845,109 rs2143968

14q22.2 53,845,573 rs2143969

14q22.2 53,846,318 rs2143972

14q22.2 53,846,722 rs12147623

14q22.2 53,847,452 rs17127455

14q22.2 53,847,495 rs7147119

14q22.2 53,854,287 rs1007141

14q22.2 53,857,291 rs10143482

14q22.2 53,862,606 rs10142602

14q22.2 53,862,815 rs7149808

14q22.2 53,863,964 rs9323262

14q22.2 53,864,913 rs10151748

14q22.2 53,866,651 rs17093094

14q22.2 53,866,718 rs10483632

14q22.2 53,868,345 rs10483633

14q22.2 53,869,789 rs10483634

14q22.2 53,870,267 rs17127477

14q22.2 53,870,595 rs10483636

14q22.2 53,871,337 rs12431978

14q22.2 53,872,350 rs9646153

14q22.2 53,872,594 rs4561356

14q22.2 53,873,515 rs10150369

14q22.2 53,873,913 rs11625167

14q22.2 53,873,970 rs10450919

14q22.2 53,875,348 rs17127485

14q22.2 53,875,469 rs17726338

14q22.2 53,875,613 rs10144925

14q22.2 53,876,666 rs11627953

14q22.2 53,877,291 rs2358662

14q22.2 53,878,203 rs7143704

14q22.2 53,880,016 rs1950198

14q22.2 53,883,773 rs8009858

14q22.2 53,885,533 rs8017164

14q22.2 53,890,332 rs761509

14q22.2 53,891,017 rs10138778

14q22.2 53,892,038 rs1950199

14q22.2 53,897,274 rs1955458

14q22.2 53,900,363 rs1955459

14q22.2 53,901,582 rs7144936

14q22.2 53,902,742 rs8018632

14q22.2 53,903,695 rs12893789

14q22.2 53,905,692 rs7147950

14q22.2 53,906,583 rs4901511

14q22.2 53,909,646 rs1950202

14q22.2 53,910,865 rs10146310

14q22.2 53,911,548 rs12888951

14q22.2 53,927,036 rs4293296

14q22.2 53,931,558 rs8007223

14q22.2 53,945,264 rs2179896

14q22.2 53,945,934 rs4251631

**CHR BP SNP**

20p12.3 6,023,178 rs2232068

20p12.3 6,031,493 rs4142219

20p12.3 6,033,006 rs11700048

20p12.3 6,033,216 rs11700084

20p12.3 6,034,772 rs6053908

20p12.3 6,037,552 rs6085405

20p12.3 6,037,797 rs6085406

20p12.3 6,041,090 rs2326719

20p12.3 6,041,177 rs16991866

20p12.3 6,044,695 rs2295435

20p12.3 6,059,569 rs6038366

20p12.3 6,062,257 rs6053925

20p12.3 6,063,581 rs6053929

20p12.3 6,066,980 rs2144936

20p12.3 6,072,113 rs1774885

20p12.3 6,074,302 rs11087720

20p12.3 6,076,201 rs947465

20p12.3 6,076,223 rs1774886

20p12.3 6,076,868 rs1778777

20p12.3 6,076,868 rs6053941

20p12.3 6,078,025 rs3897509

20p12.3 6,078,577 rs1342347

20p12.3 6,085,310 rs6076954

20p12.3 6,091,379 rs3844457

20p12.3 6,092,454 rs3852936

20p12.3 6,104,745 rs7270088

20p12.3 6,110,015 rs6085425

20p12.3 6,114,488 rs6133296

20p12.3 6,124,595 rs6053959

20p12.3 6,124,793 rs6053961

20p12.3 6,128,089 rs6085432

20p12.3 6,129,863 rs6053968

20p12.3 6,129,948 rs2021349

20p12.3 6,130,577 rs3747923

20p12.3 6,130,755 rs3747924

20p12.3 6,135,441 rs6053977

20p12.3 6,136,253 rs2799349

20p12.3 6,149,249 rs7261122

20p12.3 6,162,234 rs8121939

20p12.3 6,177,282 rs6054024

20p12.3 6,178,570 rs6133302

20p12.3 6,186,141 rs6038397

20p12.3 6,188,201 rs6038400

20p12.3 6,191,193 rs7354540

20p12.3 6,193,805 rs6117185

20p12.3 6,199,639 rs3909086

20p12.3 6,208,436 rs7362889

20p12.3 6,210,187 rs6054058

20p12.3 6,222,464 rs2326734

20p12.3 6,225,097 rs237686

20p12.3 6,234,656 rs237694

20p12.3 6,239,315 rs2326746

20p12.3 6,241,824 rs2326748

20p12.3 6,243,914 rs6139963

20p12.3 6,244,266 rs6054082

20p12.3 6,264,328 rs1571216

20p12.3 6,270,657 rs6139969

20p12.3 6,270,689 rs6054128

20p12.3 6,279,376 rs6076983

20p12.3 6,279,985 rs6085512

20p12.3 6,283,186 rs6139970

20p12.3 6,284,597 rs3920632

20p12.3 6,285,184 rs6076985

20p12.3 6,285,980 rs6038449

20p12.3 6,299,526 rs7265326

20p12.3 6,309,273 rs6085525

20p12.3 6,311,480 rs2148815

20p12.3 6,312,018 rs6038472

20p12.3 6,316,089 rs2182712

20p12.3 6,319,011 rs2209761

20p12.3 6,336,068 rs355527

20p12.3 6,352,281 rs961253

20p12.3 6,354,102 rs6085549

20p12.3 6,355,642 rs6139980

20p12.3 6,357,005 rs6085552

20p12.3 6,359,203 rs6139981

20p12.3 6,364,132 rs953420

20p12.3 6,364,883 rs7265115

20p12.3 6,366,899 rs2026168

20p12.3 6,370,001 rs6038495

20p12.3 6,370,697 rs6117260

20p12.3 6,374,137 rs6133317

20p12.3 6,376,764 rs4815900

20p12.3 6,379,267 rs6054264

20p12.3 6,379,316 rs953959

20p12.3 6,382,266 rs2225348

20p12.3 6,383,839 rs6140000

20p12.3 6,390,961 rs6077004

20p12.3 6,392,931 rs7269050

20p12.3 6,396,678 rs6117277

20p12.3 6,400,982 rs6085568

20p12.3 6,402,661 rs6054292

20p12.3 6,403,198 rs6140007

20p12.3 6,405,336 rs979548

20p12.3 6,411,095 rs13041415

20p12.3 6,411,734 rs6054304

20p12.3 6,416,261 rs10485705

20p12.3 6,416,371 rs1970008

20p12.3 6,418,805 rs6117294

20p12.3 6,421,123 rs6140010

20p12.3 6,423,234 rs6038521

20p12.3 6,426,585 rs4815908

20p12.3 6,434,648 rs6107820

20p12.3 6,435,687 rs6117303

20p12.3 6,448,296 rs6085588

20p12.3 6,457,880 rs2423161

20p12.3 6,461,935 rs2423163

20p12.3 6,467,677 rs6117325

20p12.3 6,486,532 rs1321440

20p12.3 6,496,863 rs1321443

20p12.3 6,499,421 rs2104012

20p12.3 6,507,149 rs16992376

20p12.3 6,508,776 rs6117342

20p12.3 6,509,262 rs1884898

20p12.3 6,520,014 rs2145274

20p12.3 6,521,455 rs2145275

20p12.3 6,532,604 rs6054383

20p12.3 6,539,010 rs6038556

20p12.3 6,543,646 rs4813800

20p12.3 6,544,582 rs2064857

20p12.3 6,552,972 rs13038490

20p12.3 6,560,832 rs1884897

20p12.3 6,565,959 rs6085635

20p12.3 6,568,893 rs967417

20p12.3 6,569,306 rs13042347

20p12.3 6,571,374 rs979012

20p12.3 6,574,899 rs4815914

20p12.3 6,576,901 rs6054421

20p12.3 6,579,055 rs1358805

20p12.3 6,585,234 rs984677

20p12.3 6,591,172 rs4815919

20p12.3 6,591,207 rs6054434

20p12.3 6,595,633 rs11696118

20p12.3 6,596,127 rs4815922

20p12.3 6,597,874 rs13037957

20p12.3 6,600,824 rs11087739

20p12.3 6,602,029 rs2876049

20p12.3 6,603,780 rs6077047

20p12.3 6,610,678 rs6107863

20p12.3 6,611,715 rs2143712

20p12.3 6,616,179 rs2206920

20p12.3 6,618,381 rs6054459

20p12.3 6,619,980 rs6038585

20p12.3 6,620,117 rs6107864

20p12.3 6,620,360 rs6054464

20p12.3 6,621,018 rs6054465

20p12.3 6,624,723 rs6054474

20p12.3 6,625,128 rs2206922

20p12.3 6,626,538 rs4815931

20p12.3 6,629,459 rs2038212

20p12.3 6,634,426 rs6085659

20p12.3 6,637,058 rs6054479

20p12.3 6,639,069 rs6085660

20p12.3 6,641,128 rs6085661

20p12.3 6,642,203 rs11698483

20p12.3 6,642,921 rs12106163

20p12.3 6,645,820 rs8120537

20p12.3 6,646,687 rs2326849

20p12.3 6,647,595 rs4813802

20p12.3 6,648,202 rs6054486

20p12.3 6,648,466 rs8122397

20p12.3 6,653,246 rs235763

20p12.3 6,656,948 rs2746251

20p12.3 6,658,434 rs6054494

20p12.3 6,659,863 rs235713

20p12.3 6,662,108 rs1500545

20p12.3 6,666,939 rs6054501

20p12.3 6,669,950 rs11905385

20p12.3 6,671,411 rs11906435

20p12.3 6,673,092 rs13044457

20p12.3 6,673,829 rs6085671

20p12.3 6,674,127 rs17804047

20p12.3 6,676,238 rs235721

20p12.3 6,677,212 rs11905869

20p12.3 6,677,644 rs6140076

20p12.3 6,677,789 rs6117415

20p12.3 6,688,089 rs235711

20p12.3 6,688,366 rs235710

20p12.3 6,694,498 rs1980499

20p12.3 6,704,148 rs1005464

20p12.3 6,710,719 rs235772

20p12.3 6,713,841 rs173107

20p12.3 6,714,019 rs235757

20p12.3 6,715,642 rs910141

20p12.3 6,717,533 rs235753

20p12.3 6,718,144 rs235749

20p12.3 6,718,690 rs6085681

20p12.3 6,719,370 rs6054517

20p12.3 6,720,263 rs235704

20p12.3 6,726,978 rs1157079

20p12.3 6,731,274 rs2650972

20p12.3 6,735,507 rs235716

20p12.3 6,740,145 rs235725

20p12.3 6,744,219 rs996544

20p12.3 6,746,495 rs6054529

20p12.3 6,747,833 rs6054534

20p12.3 6,755,276 rs2224191

20p12.3 6,758,998 rs372398

20p12.3 6,761,845 rs6054538

20p12.3 6,762,743 rs6107882

20p12.3 6,767,428 rs6085693

20p12.3 6,770,165 rs6038617

20p12.3 6,772,496 rs6054545

20p12.3 6,774,062 rs6038619

20p12.3 6,775,743 rs11906628

20p12.3 6,782,380 rs6038621

20p12.3 6,785,363 rs6054552

20p12.3 6,786,358 rs723511

20p12.3 6,787,006 rs16992673

20p12.3 6,787,494 rs769385

20p12.3 6,792,846 rs1392625

20p12.3 6,802,187 rs4813807

20p12.3 6,808,895 rs1028394

20p12.3 6,810,289 rs1883719

20p12.3 6,826,037 rs13044713

20p12.3 6,829,898 rs6107891

20p12.3 6,831,464 rs6054590

20p12.3 6,834,744 rs6038639

20p12.3 6,841,128 rs6038644

20p12.3 6,846,806 rs6038647

20p12.3 6,846,828 rs6038648

20p12.3 6,850,150 rs6085728

20p12.3 6,853,861 rs6085732

20p12.3 6,859,707 rs6077094

20p12.3 6,862,617 rs6140124

20p12.3 6,872,681 rs6077102

20p12.3 6,874,675 rs10485708

20p12.3 6,875,369 rs6038661

20p12.3 6,878,578 rs7263691

20p12.3 6,879,717 rs2326851

20p12.3 6,882,897 rs4815952

20p12.3 6,887,579 rs6085775

20p12.3 6,888,680 rs6077113

20p12.3 6,890,420 rs6054631

20p12.3 6,896,587 rs6085781

20p12.3 6,900,829 rs6077115

20p12.3 6,904,928 rs16992851

20p12.3 6,911,240 rs6085802

20p12.3 6,912,082 rs6085806

20p12.3 6,917,479 rs6107902

20p12.3 6,918,514 rs6107904

20p12.3 6,919,610 rs6038678

20p12.3 6,926,096 rs6085817

20p12.3 6,928,635 rs6085820

20p12.3 6,938,833 rs2149639

20p12.3 6,942,830 rs6133387

20p12.3 6,945,406 rs2209982

20p12.3 6,954,365 rs4616542

20p12.3 6,954,604 rs4616543

20p12.3 6,955,883 rs4815963

20p12.3 6,959,219 rs6117564

20p12.3 6,962,445 rs2149642

20p12.3 6,962,717 rs2183184

20p12.3 6,964,232 rs756320

20p12.3 6,975,965 rs12625876

20p12.3 6,976,859 rs6054701

20p12.3 6,977,823 rs2026056

20p12.3 6,979,190 rs4813815

20p12.3 6,982,547 rs6117583

20p12.3 6,989,513 rs2225265

20p12.3 6,995,285 rs6054709

20p12.3 6,998,017 rs3761882

20p12.3 6,998,150 rs3761884

20p12.3 7,000,333 rs6077138

20p12.3 7,005,829 rs6054715

20p12.3 7,009,833 rs7269131

20p12.3 7,015,037 rs232651

20p12.3 7,020,555 rs1005351

20p12.3 7,033,280 rs368007

20p12.3 7,042,534 rs232657

20p12.3 7,044,030 rs232656

20p12.3 7,054,353 rs1884303

20p12.3 7,056,042 rs6117640

20p12.3 7,060,386 rs6054755

20p12.3 7,067,491 rs2326896

20p12.3 7,069,672 rs6107929

20p12.3 7,077,738 rs6038724

20p12.3 7,080,239 rs1015357

20p12.3 7,085,757 rs6038729

20p12.3 7,090,997 rs719563

20p12.3 7,099,968 rs179753

20p12.3 7,112,725 rs6085916

20p12.3 7,118,080 rs4815978

20p12.3 7,120,181 rs6117672

20p12.3 7,139,569 rs6054805

20p12.3 7,139,960 rs6054806

20p12.3 7,147,413 rs724940

20p12.3 7,147,917 rs6140212

20p12.3 7,150,442 rs6054823

20p12.3 7,155,387 rs4140471

20p12.3 7,156,149 rs2207584

20p12.3 7,158,137 rs2326905

20p12.3 7,159,515 rs4813818

20p12.3 7,160,442 rs6085933

20p12.3 7,168,930 rs6085938

20p12.3 7,180,828 rs6085947

20p12.3 7,193,805 rs6117728

20p12.3 7,195,239 rs6054856

20p12.3 7,198,328 rs13045456

20p12.3 7,201,477 rs6107955

20p12.3 7,201,500 rs6117734

20p12.3 7,202,807 rs4815988

20p12.3 7,204,082 rs6085961

20p12.3 7,207,925 rs10485715

20p12.3 7,211,727 rs6117741

20p12.3 7,214,086 rs6085968

20p12.3 7,219,214 rs6054883

20p12.3 7,235,253 rs6085987

20p12.3 7,240,267 rs6077184

20p12.3 7,241,040 rs6516269

20p12.3 7,241,535 rs2423191

20p12.3 7,242,973 rs6054907

20p12.3 7,245,420 rs4813825

20p12.3 7,255,816 rs721708

20p12.3 7,265,250 rs726699

20p12.3 7,274,701 rs971226

20p12.3 7,285,884 rs16993461

20p12.3 7,290,895 rs2423214

20p12.3 7,297,731 rs2423222

20p12.3 7,318,428 rs6054963

20p12.3 7,319,819 rs6086025

20p12.3 7,322,248 rs6054965

20p12.3 7,322,894 rs6086028

20p12.3 7,326,751 rs2423248

20p12.3 7,328,416 rs6086031

20p12.3 7,328,574 rs6038803

20p12.3 7,333,248 rs2423254

20p12.3 7,347,802 rs1124635

20p12.3 7,348,195 rs16993560

20p12.3 7,352,346 rs6054992

20p12.3 7,354,438 rs2207717

20p12.3 7,372,609 rs6038832

20p12.3 7,372,828 rs6038833

20p12.3 7,383,615 rs17399484

20p12.3 7,389,840 rs971103

20p12.3 7,401,335 rs4813827

20p12.3 7,405,161 rs6140294

20p12.3 7,406,007 rs6107991

20p12.3 7,410,555 rs2103866

20p12.3 7,430,971 rs6055065

20p12.3 7,436,279 rs6055068

20p12.3 7,440,033 rs6086074

20p12.3 7,443,442 rs6077218

20p12.3 7,444,565 rs6086075

20p12.3 7,445,003 rs7263704

20p12.3 7,445,194 rs6055075

20p12.3 7,447,209 rs6038861

20p12.3 7,448,401 rs6055076

20p12.3 7,449,871 rs6055078

20p12.3 7,460,152 rs6055085

20p12.3 7,467,612 rs17310112

20p12.3 7,478,600 rs6055103

20p12.3 7,478,600 rs7347783

20p12.3 7,485,811 rs13037510

20p12.3 7,489,572 rs6086103

20p12.3 7,489,572 rs7353305

20p12.3 7,493,361 rs4816009

20p12.3 7,499,917 rs1022632

20p12.3 7,500,264 rs1022631

20p12.3 7,500,504 rs6108011

20p12.3 7,518,459 rs2014101

20p12.3 7,519,155 rs6133438

20p12.3 7,529,461 rs6140334

20p12.3 7,529,461 rs8184119

20p12.3 7,537,465 rs6117882

20p12.3 7,542,246 rs6117883

20p12.3 7,543,488 rs6140342

20p12.3 7,544,690 rs6077231

20p12.3 7,550,235 rs6140348

20p12.3 7,552,121 rs12151931

20p12.3 7,556,657 rs6108026

20p12.3 7,562,902 rs2206394

20p12.3 7,563,276 rs2223530

20p12.3 7,566,077 rs6086132

20p12.3 7,572,747 rs6086143

20p12.3 7,573,506 rs6140362

20p12.3 7,575,224 rs2050081

20p12.3 7,575,294 rs6108030

20p12.3 7,587,078 rs6086150

20p12.3 7,587,093 rs2223534

20p12.3 7,592,140 rs6055239

20p12.3 7,596,350 rs6133451

20p12.3 7,601,778 rs6133455

20p12.3 7,602,897 rs6055249

20p12.3 7,604,614 rs4816018

20p12.3 7,607,991 rs6077242

20p12.3 7,610,888 rs4140533

20p12.3 7,612,282 rs6055253

20p12.3 7,614,258 rs6055254

20p12.3 7,625,668 rs969111

20p12.3 7,628,047 rs6133460

20p12.3 7,628,399 rs6108042

20p12.3 7,629,358 rs1008096

20p12.3 7,629,631 rs4258870

20p12.3 7,635,324 rs761007

20p12.3 7,635,801 rs729943

20p12.3 7,636,055 rs729944

20p12.3 7,637,193 rs6140393

20p12.3 7,640,889 rs6133475

20p12.3 7,641,459 rs6038934

20p12.3 7,648,108 rs2294551

20p12.3 7,648,582 rs7263060

20p12.3 7,652,212 rs6133481

20p12.3 7,652,428 rs6108046

20p12.3 7,685,540 rs6140410

20p12.3 7,687,376 rs6140412

20p12.3 7,690,500 rs6140414

20p12.3 7,700,366 rs6077251

20p12.3 7,704,027 rs2092380

20p12.3 7,725,476 rs6140425

20p12.3 7,747,315 rs2423268

20p12.3 7,758,099 rs10485717

20p12.3 7,763,941 rs6086243

20p12.3 7,766,261 rs2423290

20p12.3 7,767,768 rs2423294

20p12.3 7,776,186 rs11698712

20p12.3 7,780,380 rs6077276

20p12.3 7,784,714 rs7269743

20p12.3 7,790,234 rs4239773

20p12.3 7,797,927 rs2205824

20p12.3 7,799,039 rs6086260

20p12.3 7,799,422 rs6117972

20p12.3 7,804,704 rs6140453

20p12.3 7,817,025 rs2294306

20p12.3 7,821,112 rs2423322

20p12.3 7,821,818 rs6133511

20p12.3 7,824,255 rs6117980

20p12.3 7,825,416 rs1569454
