# Supplementary material for: Multiple Common Susceptibility Variants near BMP Pathway Loci GREM1, BMP4, and BMP2 Explain Part of the Missing Heritability of Colorectal Cancer
Source: PLoS Genet. 2011 Jun 2;7(6):e1002105. doi: 10.1371/journal.pgen.1002105 (PMC3107194; doi:10.1371/journal.pgen.1002105)
Supplement: Table S5 — Additional BMP pathway genes around which tagSNP associations with CRC were analysed. (DOCX) [file pgen.1002105.s011.docx]

*Supplemental Table 5. Additional BMP pathway genes around which tagSNP associations with CRC were analysed.*

BMPR1A

BMPR1B

BMPR2

GREM1

GREM2

NOG

CER1

DAND5/GREM3

NBL1

CHRD

FST

FSTL1

FSTL3

FSTL4

FSTL5

BMP2

BMP3

BMP4

BMP5

BMP6

BMP7

BMP8A

BMP8B

BMP9/GDF2

BMP10

BMP11/GDF11

SMAD1

SMAD5

SMAD9 (SMAD8)

SMAD4

SMAD6

SMAD7

BAMBI

SMURF1

SYCP1

SYCP2

SYCP3
