# Supplementary material for: Multiple Common Susceptibility Variants near BMP Pathway Loci GREM1, BMP4, and BMP2 Explain Part of the Missing Heritability of Colorectal Cancer
Source: PLoS Genet. 2011 Jun 2;7(6):e1002105. doi: 10.1371/journal.pgen.1002105 (PMC3107194; doi:10.1371/journal.pgen.1002105)
Supplement: Text S1 — Consortium co-authors. (DOCX) [file pgen.1002105.s012.docx]

**Supplemental Material: Consortium Co-Authors**

The CORGI Consortium comprises: Eamonn Maher, Dept. of Clinical Genetics, University of Birmingham, UK; Gareth Evans, Dept. of Clinical Genetics, University of Manchester, UK; Lisa Walker and Dorothy Halliday, Oxford Regional Genetics Service, Churchill Hospital, Oxford, UK; Anneke Lucassen, Wessex Regional Genetics Service, Princess Anne Hospital, Southampton, UK; Joan Paterson, Anglia Regional Genetics Service, Addenbrooke’s Hospital, Cambridge, UK; Shirley Hodgson and Tessa Homfray, South-West Thames Regional Genetics Service, St George’s Hospital, Tooting, London, UK; Lucy Side, North-East Thames Regional Genetics Service, Great Ormond Street Hospital, London, UK; Louise Izatt, South-East Thames Regional Genetics Service, Guy’s Hospital, London, UK; Alan Donaldson and Susan Tomkins, South-West Regional Genetics Service, Bristol, UK; Patrick Morrison, Northern Ireland Regional Genetics Service, City Hospital, Belfast, UK; Carole Brewer, South-West Regional Genetics Service, Royal Devon and Exeter Hospital, Exeter, UK; Alex Henderson, Northern Regional Genetics Service, International Centre for Life, Newcastle, UK; Rosemarie Davidson and Victoria Murday, West of Scotland Regional Genetics Service, Yorkhill Hospital, Glasgow, UK; Jaqueline Cook, Sheffield Regional Genetics Service, Children’s Hospital, Sheffield, UK; Neva Haites, North of Scotland Regional Genetics Service, Foresterhill Hospital, Aberdeen, UK; Timothy Bishop and Eamonn Sheridan, Yorkshire Regional Genetics Service, St James’s Hospital, Leeds, UK; Andrew Green, Republic of Ireland Genetics Service, Our Lady’s Hospital for Sick Children, Dublin, Republic of Ireland; Christopher Marks, Sue Carpenter and Mary Broughton, The Royal Surrey County Hospital, Egerton Road, Guildford, Surrey; Lynn Greenhalge, Department of Clinical Genetics, Royal Liverpool Children's Hospital, Eaton Road, Alderhay, Liverpool; Mohnish Suri,Department of Clinical Genetics, City Hospital, Hucknall Road, Nottingham

COGENT comprises I Tomlinson, M Dunlop, H Campbell, B Zanke, S Gallinger, T Hudson, T Koessler, P D Pharoah, I Niittymäki, S Tuupanen, L A Aaltonen, K Hemminki, A Lindblom, A Försti, O Sieber, L Lipton, T van Wezel, H Morreau, J T Wijnen, P Devilee, K Matsuda, Y Nakamura, S Castellví-Bel, C Ruiz-Ponte, A Castells, A Carracedo, JWC Ho, P Sham, P Vodicka, H Brenner, J Hampe, C Schafmayer, J Tepel, S Schreiber, H Völzke, MM Lerch, CA Schmidt, S Buch, V Moreno, C M Villanueva, P Peterlongo, P Radice, Li Li, MM Echeverry, M Bohorquez, A Velez, L Carvajal-Carmona, R Scott, S Penegar, P Broderick, A Tenesa and R S Houlston.

Members of the EPICOLON Consortium (Gastrointestinal Oncology Group of the Spanish Gastroenterological Association): Hospital 12 de Octubre, Madrid: Juan Diego Morillas (local coordinator), Raquel Muñoz, Marisa Manzano, Francisco Colina, Jose Díaz, Carolina Ibarrola, Guadalupe López, Alberto Ibáñez; Hospital Clínic, Barcelona: Antoni Castells (local coordinator), Virgínia Piñol, Sergi Castellví-Bel, Francesc Balaguer, Victoria Gonzalo, Teresa Ocaña, María Dolores Giráldez, Maria Pellisé, Anna Serradesanferm, Leticia Moreira, Miriam Cuatrecasas, Josep M. Piqué; Hospital Clínico Universitario, Zaragoza: Ángel Lanas (local coordinator), Javier Alcedo, Javier Ortego; Hospital Cristal-Piñor, Complexo Hospitalario de Ourense: Joaquin Cubiella (local coordinator), Mª Soledad Díez, Mercedes Salgado, Eloy Sánchez, Mariano Vega; Hospital del Mar, Barcelona: Montserrat Andreu (local coordinator), Anna Abuli, Xavier Bessa, Mar Iglesias, Agustín Seoane, Felipe Bory, Gemma Navarro, Beatriz Bellosillo; Josep Mª Dedeu, Cristina Álvarez, Begoña Gonzalez; Hospital San Eloy, Baracaldo and Hospital Donostia, CIBERehd, University of Country Basque, San Sebastián: Luis Bujanda (local coordinator) Ángel Cosme, Inés Gil, Mikel Larzabal, Carlos Placer, María del Mar Ramírez, Elisabeth Hijona, Jose M. Enríquez-Navascués y Jose L. Elosegui; Hospital General Universitario de Alicante: Artemio payá (EPICOLON I local coordinator), Rodrigo jover (EPICOLON II local coordinator), Cristina Alenda, Laura Sempere, Nuria Acame, Estefanía Rojas, Lucía Pérez-Carbonell; Hospital General de Granollers: Joaquim Rigau (local coordinator), Ángel Serrano, Anna Giménez; Hospital General de Vic: Joan Saló (local coordinator), Eduard Batiste-Alentorn, Josefina Autonell, Ramon Barniol; Hospital General Universitario de Guadalajara and Fundación para la Formación e Investigación Sanitarias Murcia: Ana María García (local coordinator), Fernando Carballo, Antonio Bienvenido, Eduardo Sanz, Fernando González, Jaime Sánchez, Akiko Ono; Hospital General Universitario de Valencia: Mercedes Latorre (local coordinator), Enrique Medina, Jaime Cuquerella, Pilar Canelles, Miguel Martorell, José Ángel García, Francisco Quiles, Elisa Orti; CHUVI-Hospital Meixoeiro, Vigo: EPICOLON I, Juan Clofent (local coordinator), Jaime Seoane, Antoni Tardío, Eugenia Sanchez. EPICOLON II, Mª Luisa de Castro (local coordinator), Antoni Tardío, Juan Clofent , Vicent Hernández; Hospital Universitari Germans Trias i Pujol, Badalona and Section of Digestive Diseases and Nutrition, University of Illinois at Chicago, IL, USA: Xavier Llor (local coordinator), Rosa M. Xicola, Marta Piñol, Mercè Rosinach, Anna Roca, Elisenda Pons, José M. Hernández, Miquel A. Gassull; Hospital Universitari Mútua de Terrassa: Fernando Fernández-Bañares (local coordinator), Josep M. Viver, Antonio Salas, Jorge Espinós, Montserrat Forné, Maria Esteve; Hospital Universitari Arnau de Vilanova, Lleida: Josep M. Reñé (local coordinator), Carmen Piñol, Juan Buenestado, Joan Viñas; Hospital Universitario de Canarias: Enrique Quintero (local coordinator), David Nicolás, Adolfo Parra, Antonio Martín; Hospital Universitario La Fe, Valencia: Lidia Argüello (local coordinator), Vicente Pons, Virginia Pertejo, Teresa Sala; Hospital Sant Pau, Barcelona: Dolors Gonzalez (local coordinator) Eva Roman, Teresa Ramon, Maria Poca, Mª Mar Concepción, Marta Martin, Lourdes Pétriz; Hospital Xeral Cies, Vigo: Daniel Martinez (local coordinator); Fundacion Publica Galega de Medicina Xenomica (FPGMX), CIBERER, Genomic Medicine Group-University of Santiago de Compostela, Santiago de Compostela, Galicia, Spain: Angel Carracedo (local coordinator), Clara Ruiz-Ponte, Ceres Fernández-Rozadilla, Mª Magdalena Castro; Hospital Universitario Central de Asturias: Sabino Riestra (local coordinator), Luis Rodrigo; Hospital de Galdácano, Vizcaya: Javier Fernández (local coordinator), Jose Luis Cabriada; Fundación Hospital de Calahorra (La Rioja)La Rioja: Luis Carreño (local coordinator), Susana Oquiñena, Federico Bolado.
